# Integrating petrophysical characterization and reservoir evaluation of the Kafr ELSheikh formation in Sapphire field, Nile Delta, Egypt

**DOI:** 10.1038/s41598-025-88056-7

**Published:** 2025-02-07

**Authors:** Naiem Souilam Sobih, Ahmed Ahmed El Nagar, Ali Elsayed Abbas, Samir Shehab

**Affiliations:** 1 Geological and Geophysical Department, Faculty of Petroleum and Mining Engineering, Suez University, Al-Galaa Street, Suez, Egypt; 2 Geology Department, Faculty of Science, Al-Azhar University, Old Cairo, Cairo, Egypt

**Keywords:** Reservoir Petrophysics, Sapphire Field Reservoir Development, Kafr ElSheikh Formation, Gas potential Assessment, Environmental sciences, Solid Earth sciences, Geophysics

## Abstract

This study aims to integrate petrophysical characterization and reservoir evaluation for optimizing field development. The focus is on the Kafr Elsheikh formation, using iso-parametric maps to analyze key reservoir parameters such as effective porosity, shale content, net-pay thickness, permeability, hydrocarbon saturation, and water saturation across lateral sections. Petrophysical characterization is essential for understanding the reservoir quality and productivity potential. The study evaluates four wells—Sapphire 1, Sapphire-Da, Sapphire-Deep 1, and Sapphire-Dh—using well logs, including gamma ray, caliper, density, neutron, and resistivity, recorded in Las files. These wells are crucial for assessing the reservoir potential and development path of the Sapphire Field. Findings reveal that the Kafr Elsheikh formation has significant reservoir potential, with effective porosity ranging from 14 to 34%, water saturation between 15% and 45%, gas saturation from 55 to 85%, and net-pay thickness from 0.46 m to 23.8 m. Lithological analysis shows a composition of sandstone and shale within the formation. The developmental trajectory of Sapphire Field indicates increases in effective porosity and hydrocarbon saturation towards the central and northwest-southeast directions. Sapphire Da is identified as the most promising well, with 85% saturation, 29% effective porosity, and a net-pay thickness of 14.04 m. The study underscores the hydrocarbon reserves in the Kafr Elsheikh formation and advocates for further exploration and development wells to enhance productivity. Insights from iso-parametric maps are vital for future exploration and development within the Sapphire Field.

## Introduction

Petrophysical characterization and reservoir evaluation are critical for hydrocarbon exploration and development, providing insights into reservoir quality and productivity potential. The Nile Delta is a prolific hydrocarbon province, yet many fields, including Sapphire Field, remain underexplored in terms of petrophysical assessment.

Problem Statements:

- Limited integration of petrophysical data has hindered effective reservoir evaluation.
- Uncertainty in lateral reservoir heterogeneity complicates recovery strategies.
- Underutilization of well-log data reduces reservoir model accuracy.
- Minimal comparative studies with neighboring fields limit regional understanding.

**Fig. 1:**
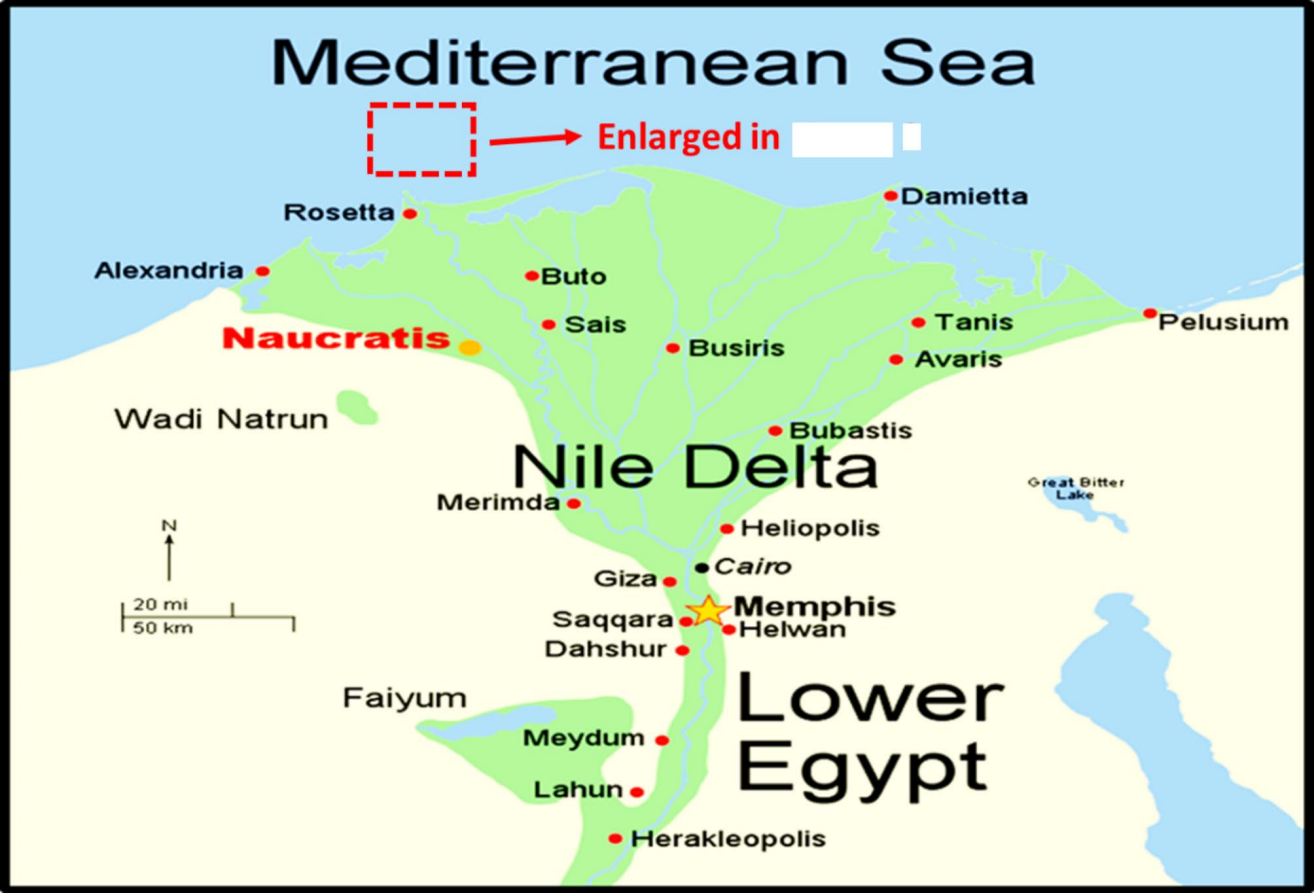
Map for Nile Delta^15^.

**Fig. 2:**
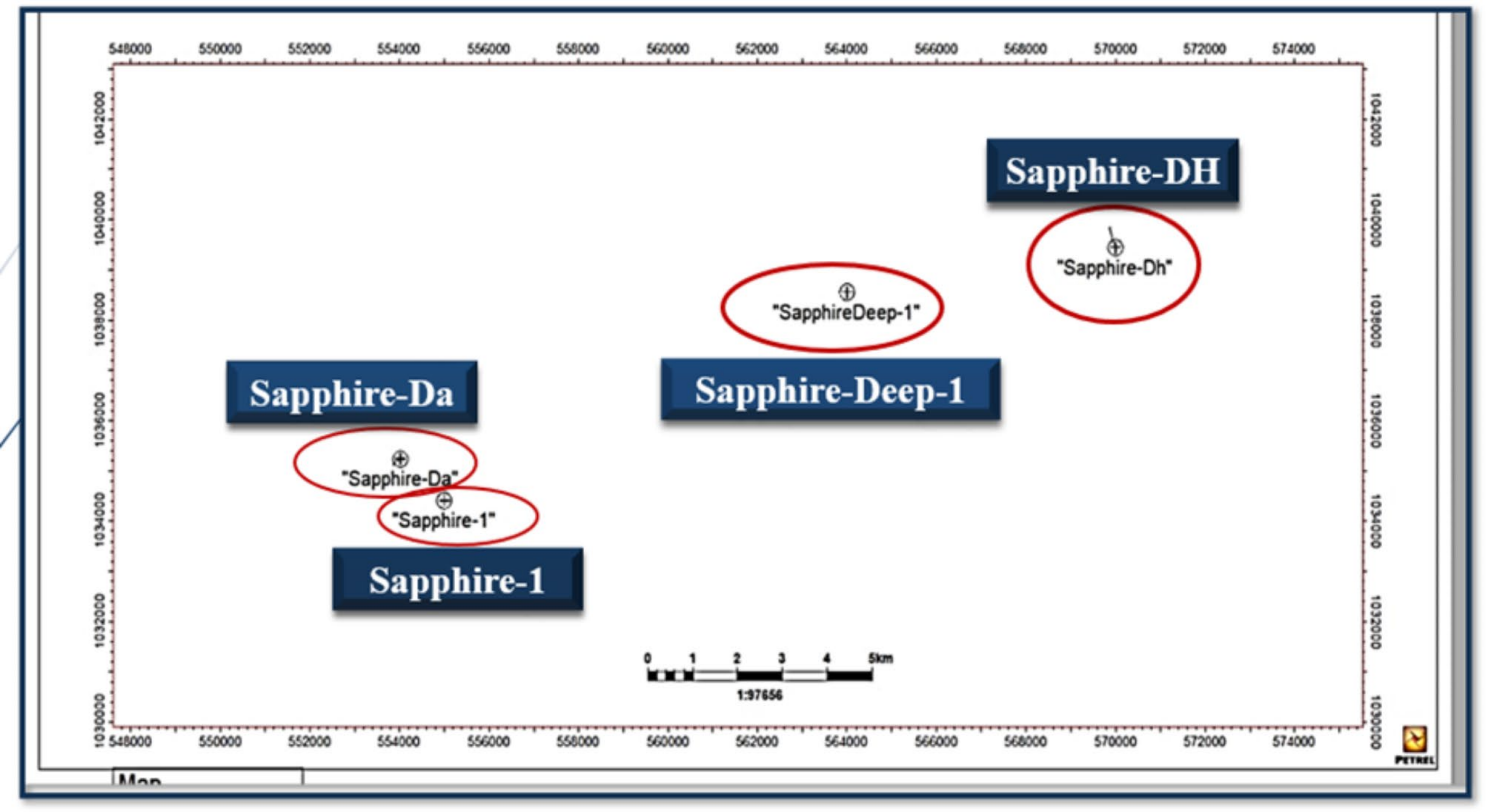
Location Map of Sapphire Field, created by me using Petrel 2017 software developed by Schlumberger.

**Fig. 3:**
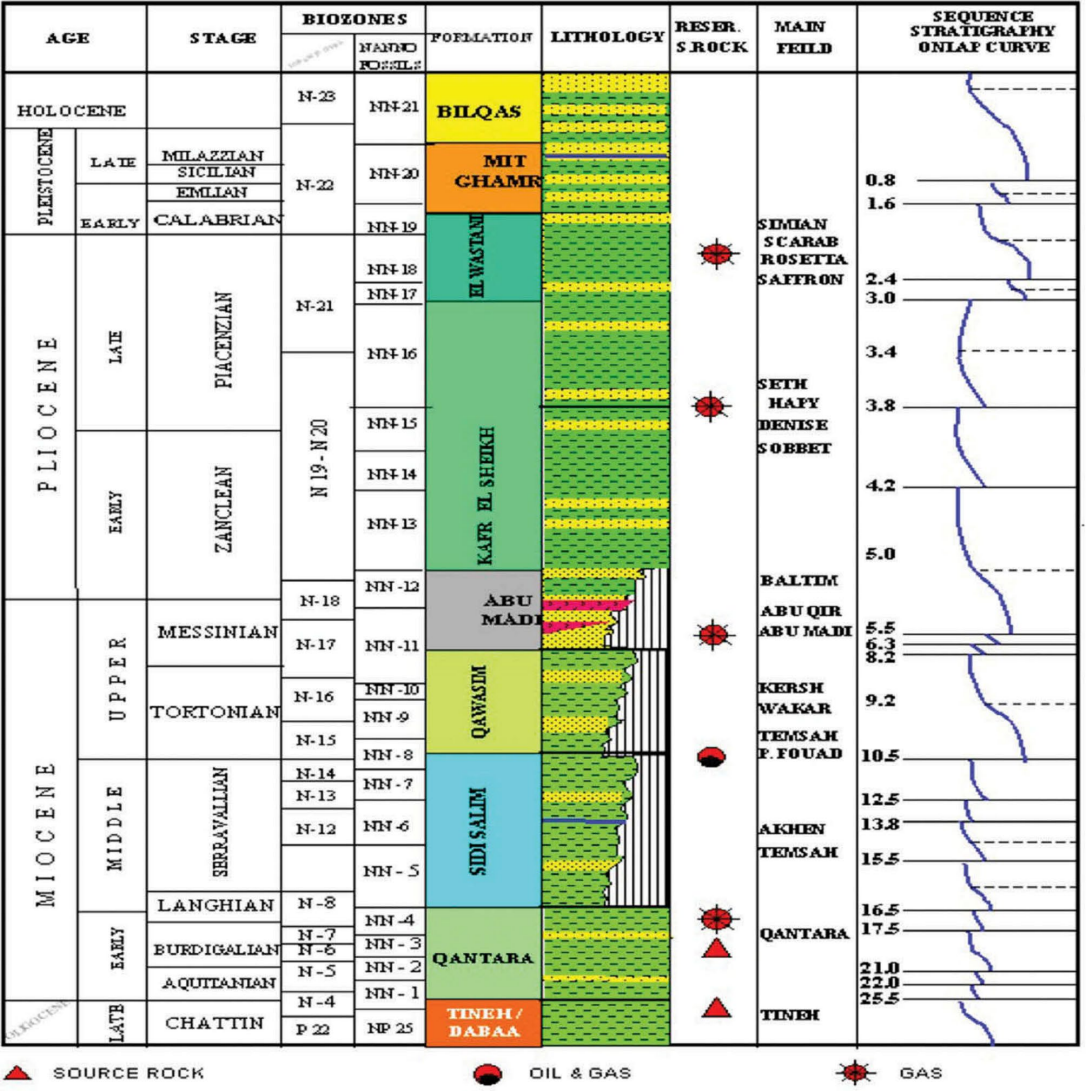
A lithostratigraphic column illustrating the general rock layers of the sapphire field within our study area^2,12^.

**Fig. 4:**
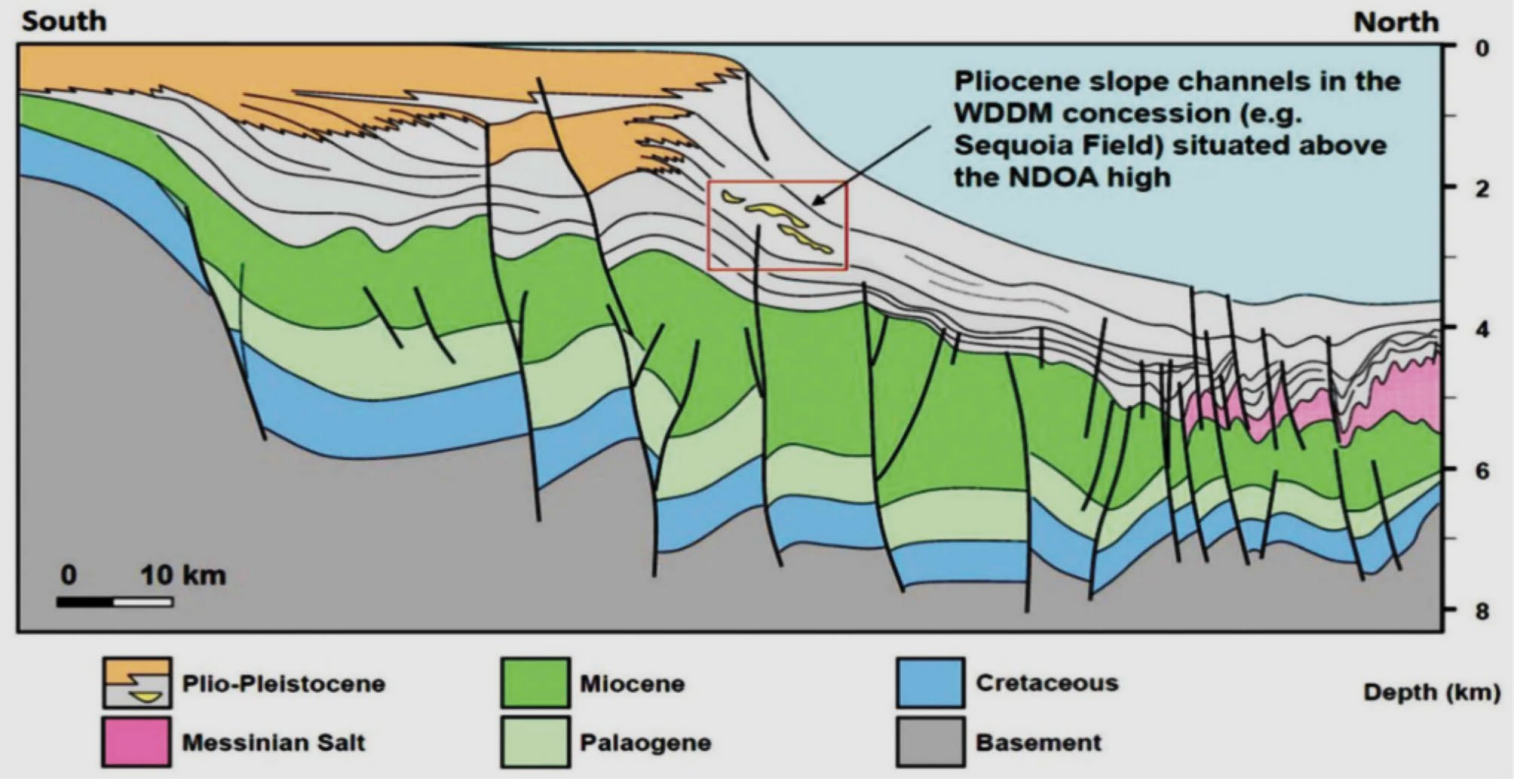
Setting of the Tectono-Stratigraphic Nile Delta^6^.

**Fig. 5:**
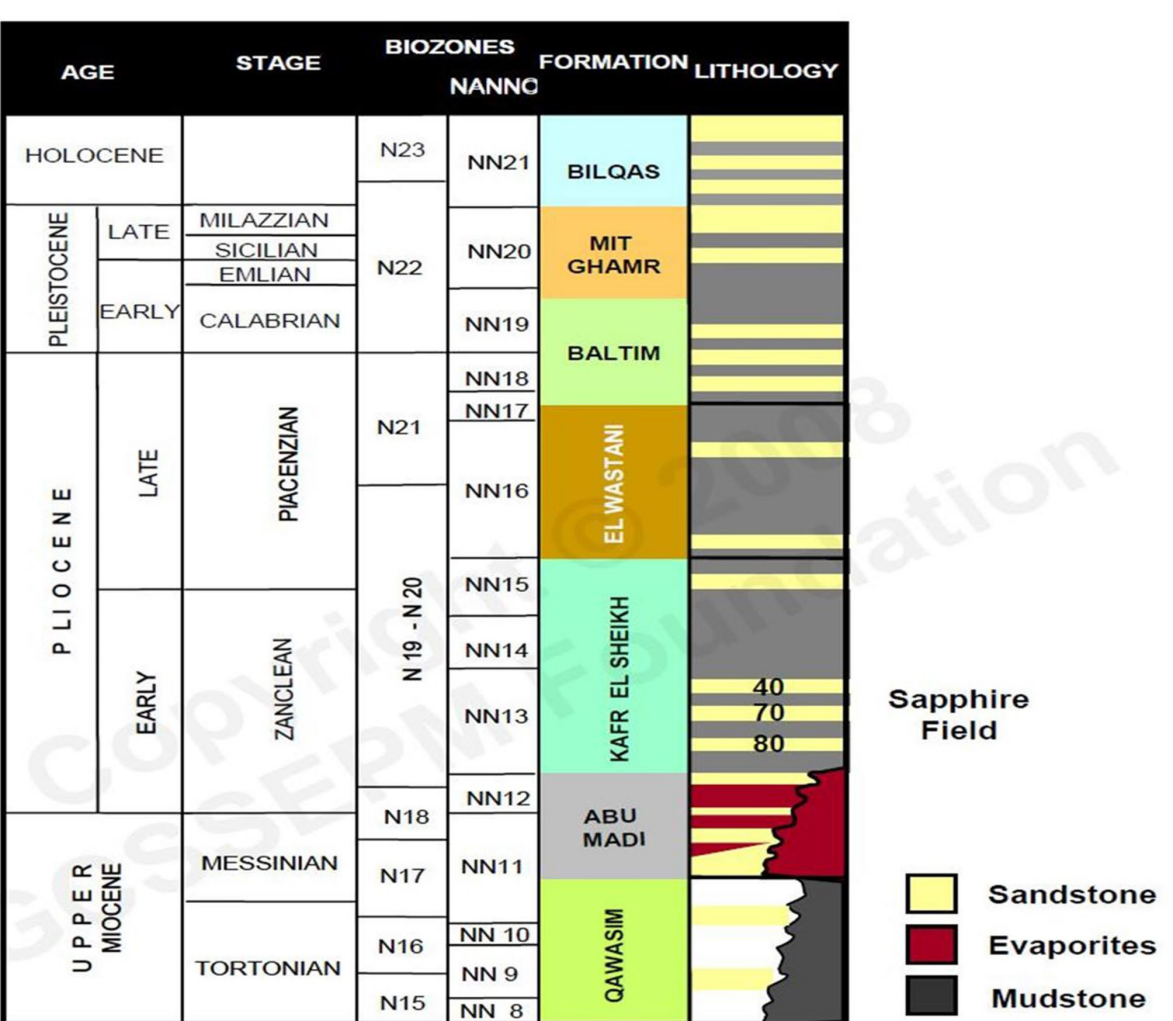
Generalized stratigraphic column including sapphire field (by BG, 2004).

The Nile Delta, shaped like the Greek letter “Delta,” spans approximately 60,000 square kilometers, both offshore and onshore (Fig. 1). The Sapphire field is located 90 km offshore Alexandria, within the Northwest Delta Deep Marine (WDDM) concession^1^, Hassan M. El Shayeb et al.^2,3^. The field extends beyond the boundary separating the Rosetta and West Delta Deep Marine concessions. Gas has been discovered in Pliocene sandstones, covering an area of approximately 90 km². The reservoir consists of sandstones, mudstones, and siltstones arranged in an upward profile. This study examines four wells in the Sapphire Field: Sapphire-1, Sapphire-Da, Sapphire-Dh, and Sapphire-Deep1 (Fig. 2). The sedimentary succession in the Nile Delta spans from the Lower Miocene to the present (Fig. 3). The Plio-Pleistocene Unit dominates the delta and includes formations such as Bilqas, Mit Ghamr, and El Wastani. Kafr Elsheikh Formation is also part of this unit, with shale being the primary lithology. The Western Desert of Egypt is a fault-bounded region with three major fault trends. These trends are described by^4,5^, and Hassan M. El Shayeb et al. (2020). The Rosetta fault is the largest, likely forming during Pliocene deposition as a SW-NE oriented extensional to strike-slip fault. This fault is supported by data from^6^ and Hassan M. El Shayeb et al.^3^ (Fig. 4). The Baltim Eastern and Northern offshore gas fields contain high gas condensate accumulations. The Abu Madi and Qawasim Formations were evaluated using wireline logs, core data, and pressure data. Petrophysical analysis revealed good porosity and high permeability, aiding in exploration site identification^7^.The lithology of the Sapphire Field mainly consists of shale interspersed with sandstone layers (Fig. 5). Shales dominate the lower portions, while sandstones appear in the upper sequences^8^. This lithological distribution influences porosity and permeability variations within the reservoir. Shale and sandstone layers play a critical role in fluid flow and hydrocarbon recovery. This study aims to perform detailed petrophysical analysis of the Kafr Elsheikh Formation. It also assesses reservoir quality in the Sapphire Field to enhance field development strategies.

## Methodology

The potential for petroleum in the clastic reservoir rock of the Early to Middle Pliocene Kafr Sheikh Formation was assessed using four wells: Sapphire-1, Sapphire-Da, Sapphire-Dh, and Sapphire-Deep1. The evaluation was based on petrophysical analysis of specific intervals in each well. This involved interpreting lithology through cross plots and contouring various petrophysical properties. The vertical distribution of these properties was mapped. The methodology consists of two main components: Petrophysical Characterization and Reservoir Evaluation. The study integrates gamma ray, density, neutron, and resistivity logs. It evaluates effective porosity, permeability, shale content, gas saturation, and net pay thickness. The Interactive Petrophysics (Techlog) 3.4 and Petrel 2017 software, developed by Schlumberger, were used for all petrophysical calculations and to create all figures in this study.

### Petrophysical characterization

#### Determination of Shale volume (V_Sh_)

The calculation of the Volume of Shale (V_sh_) is crucial in distinguishing between reservoir and non-reservoir rocks^9^. The shale volume is determined using the following equation:

1 $$\:{\text{I}}_{\text{G}\text{R}}=\frac{{\text{G}\text{R}}_{\text{l}\text{o}\text{g}}-{\text{G}\text{R}}_{\text{m}\text{i}\text{n}}}{{\text{G}\text{R}}_{\text{m}\text{a}\text{x}}-{\text{G}\text{R}}_{\text{m}\text{i}\text{n}}}$$

Where I_GR_ is the Gamma Ray Index that can be adjusted to V_sh_ using the Dresser Atlas chart^10^. GR_log_ is the gamma ray reading of the formation; GR_min_ is the minimum gamma ray; and GR_max_ is the maximum gamma ray.

#### Determination of formation porosity (Φ)

Neutron, density, and sonic logs are used in well logging to determine porosity. Neutron logs are useful for clean, unconsolidated sands. Density logs help determine porosity in consolidated rocks. Sonic logs measure the time it takes for sound waves to travel through the rock. This time is related to the rock’s density and porosity. Together, these logs provide a comprehensive picture of the reservoir’s porosity and fluid storage capacity.

2 $$\:{\varvec{\Phi\:}}_{\mathbf{t}}=\frac{{{\uprho\:}}_{\text{m}\text{a}}-{{\uprho\:}}_{\text{l}\text{o}\text{g}}}{{{\uprho\:}}_{\text{m}\text{a}}-{{\uprho\:}}_{\text{f}\text{l}}}$$

Where:

Φ = formation porosity, fraction.

$\:{\varvec{\rho\:}}_{\varvec{m}\varvec{a}}$=Density of the rock matrix, g/$\:{\text{c}\text{m}}^{3}$.

$\:{\varvec{\rho\:}}_{\varvec{f}\varvec{l}}$= Density of saturation fluid, g/$\:{\text{c}\text{m}}^{3}$.

$\:{\varvec{\rho\:}}_{\varvec{l}\varvec{o}\varvec{g}}$ = Density log reading (bulk density), g/$\:{\text{c}\text{m}}^{3}$.

The mixture’s density of mud filtrate and formation fluid is represented by the apparent fluid density. Due to the oil and mud concentration in the formation fluid, which is used in drilling, the fluid density is equal to $\:{\varvec{\rho\:}}_{\varvec{f}\varvec{l}}$ = 0.9 g/ $\:{\text{c}\text{m}}^{3}$.

3 $$\:{\varvec{\Phi\:}}_{\mathbf{e}\mathbf{f}\mathbf{f}}=\frac{{\varvec{\uprho\:}}_{\mathbf{m}\mathbf{a}}-{\varvec{\uprho\:}}_{\mathbf{b}}-{\text{V}}_{\text{c}\text{l}}\times\:({\varvec{\uprho\:}}_{\mathbf{m}\mathbf{a}}-{\varvec{\uprho\:}}_{\mathbf{c}\mathbf{l}\mathbf{a}\mathbf{y}})}{{\varvec{\uprho\:}}_{\mathbf{m}\mathbf{a}}-{\varvec{\uprho\:}}_{\mathbf{f}\mathbf{l}}}$$

Where$\:{\varvec{\Phi\:}}_{\varvec{e}\varvec{f}\varvec{f}}$ is the shale-corrected density porosity, $\:{\mathbf{V}}_{\varvec{c}\varvec{l}}$ is volume of shale and ρsh is density of shale, $\:{\varvec{\rho\:}}_{\varvec{m}\varvec{a}}$ is density of rock matrix and $\:{\varvec{\rho\:}}_{\varvec{f}\varvec{l}}$ is density of fluid.

#### Determination of water saturation (Sw)

The primary focus of fluid saturation identification is to differentiate between water and hydrocarbons. Hydrocarbons are divided into mobile and residual types^9^. The most critical petrophysical characteristic for evaluating a reservoir is water saturation (Sw)^9^. Various equations calculate water saturation, but Archie’s Indonesian equation is the most commonly used and essential.

4 $$\:{\mathbf{S}}_{\mathbf{W}\mathbf{i}\mathbf{n}\mathbf{d}\mathbf{o}}={\left\{\frac{\sqrt{\frac{1}{{\text{R}}_{\text{t}}}}}{\left(\frac{{{\text{V}}_{\text{s}\text{h}}}^{\left(1-0.5{\text{V}}_{\text{s}\text{h}}\right)}}{\sqrt{{\text{R}}_{\text{s}\text{h}}}}\right)+\frac{{{\varnothing}}^{\text{m}/2}}{{\left({\varnothing}\text{*}{\text{R}}_{\text{w}}\right)}^{0.\text{s}}}}\right\}}^{\left(2/\text{n}\right)}$$

Where; $\:{\varvec{S}}_{\varvec{w}\varvec{i}\varvec{n}\varvec{d}\varvec{o}}$= water saturation, fraction, $\:{\varvec{R}}_{\varvec{s}\varvec{h}}$= the resistivity of a thick shale unit, **R**_**t**_ = the true resistivity of the uninvaded formation, $\:{\varvec{V}}_{\varvec{s}\varvec{h}}$=volume of shale, $\:\varvec{\Phi\:}$ = porosity, fraction, $\:{\varvec{R}}_{\varvec{w}}$ = resistivity of formation water, ohm, **m** = cementation factor.

#### Determination of permeability (K)

Permeability (K) refers to a porous medium’s ability to transmit fluids. It is calculated using Coates Eq. (5) from Schlumberger’s Log Interpretation^11^. Water saturation calculated above the free water level is considered irreducible water saturation.

5 $$\:\mathbf{k}={\mathbf{k}}_{\mathbf{c}}\times\:{{\varvec{\Phi\:}}_{\mathbf{e}\mathbf{f}\mathbf{f}}}^{4}\times\:{\left(\frac{1-\:{\mathbf{S}}_{\mathbf{W}\mathbf{i}\mathbf{r}\mathbf{r}}}{{\mathbf{S}}_{\mathbf{W}\mathbf{i}\mathbf{r}\mathbf{r}}}\right)}^{2}$$

Where:

**(**$\:{\varvec{S}}_{\varvec{w}\varvec{i}\varvec{r}\varvec{r}}$**) =** irreducible water saturation, **(**$\:{\varvec{\Phi\:}}_{\varvec{e}\varvec{f}\varvec{f}}$**) =** effective porosity, and **(**$\:{\varvec{k}}_{\varvec{c}}$**) =** permeability coefficient.

#### Determination of gas saturation (Sg)

Through the use of effective porosity.

$$\:{\mathbf{S}}_{\mathbf{g}}$$ = (1 - $$\:{\mathbf{S}}_{\mathbf{W}}$$) (6)

Where: **(**$\:{\varvec{S}}_{\varvec{g}}$**)** Hydrocarbon saturation, **(**$\:{\mathbf{S}}_{\varvec{w}}$**)** water saturation.

#### Determination of netpay thickness

The netpay thickness is determined by taking the log average of the pay net flag.

### Reservoir evaluation

Reservoir evaluation focuses on analyzing the lateral variations of petrophysical properties within the reservoir. This step involves creating iso-parametric maps to assess the hydrocarbon potential across different sections of the field. The maps are generated by integrating key petrophysical parameters, such as porosity, permeability, and saturation, to highlight zones of high hydrocarbon concentration.The study also evaluates the distribution of these properties vertically and laterally within the formation. This is done by mapping the spatial variability of effective porosity, gas saturation, water saturation, and permeability, which helps identify optimal areas for further exploration and development. To ensure the accuracy of the findings, the results from the four wells are cross-referenced with regional trends in the Nile Delta. Despite the limited number of wells, these wells are considered representative of the reservoir’s characteristics and provide a reliable basis for the evaluation of the Sapphire Field’s hydrocarbon potential. The analysis also identifies the areas with the highest reservoir quality and production potential, which can guide future drilling and field development plans. These insights are crucial for optimizing hydrocarbon recovery and improving the economic feasibility of the field.

## Results and discussion

Based on wireline logging data.

### Petrophysical assessment

The petrophysical evaluation of the Kafr El Sheikh Formation involved analyzing porosity, permeability, water saturation, hydrocarbon saturation, and shale content. This analysis used well logging data from four wells in the Sapphire Field: Sapphire-1, Sapphire-Da, Sapphire-Dh, and Sapphire-Deep1.

#### Petrophysical interpretation log of Sapphire wells

**Fig. 6:**
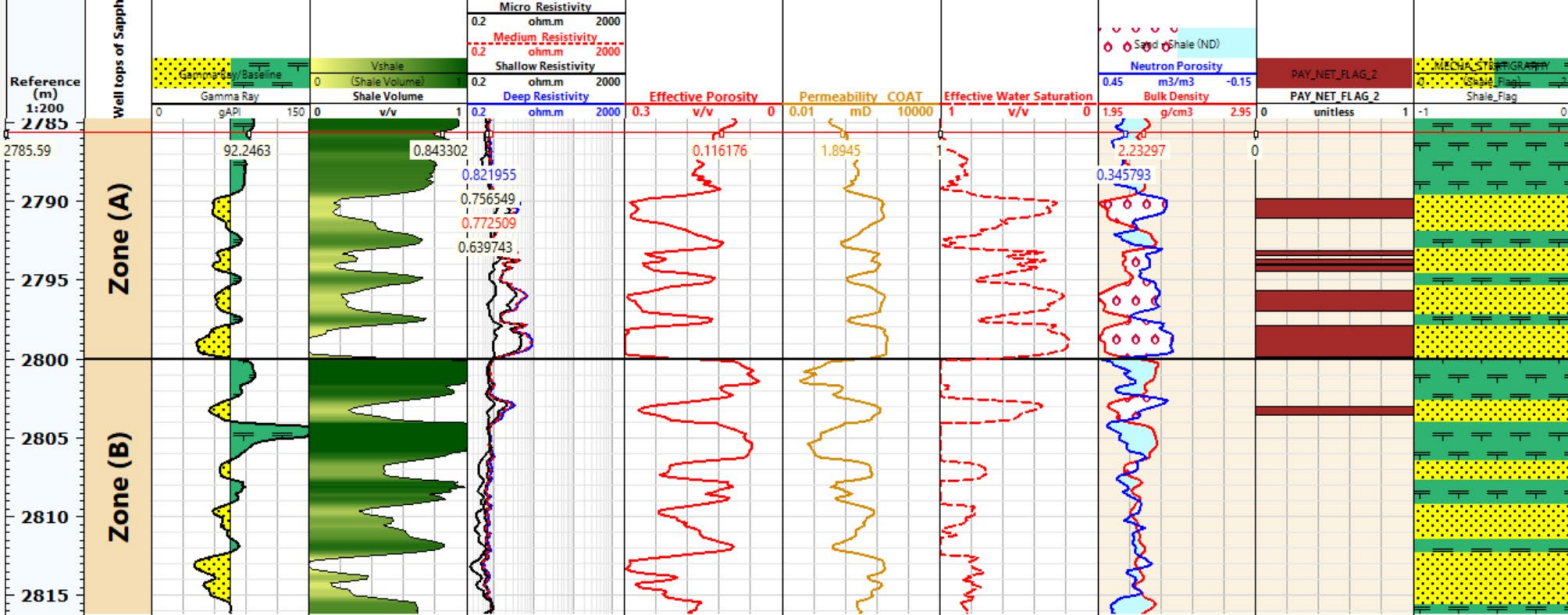
Petrophysical Interpretation log of Sapphire-1 well.

**Fig. 7:**
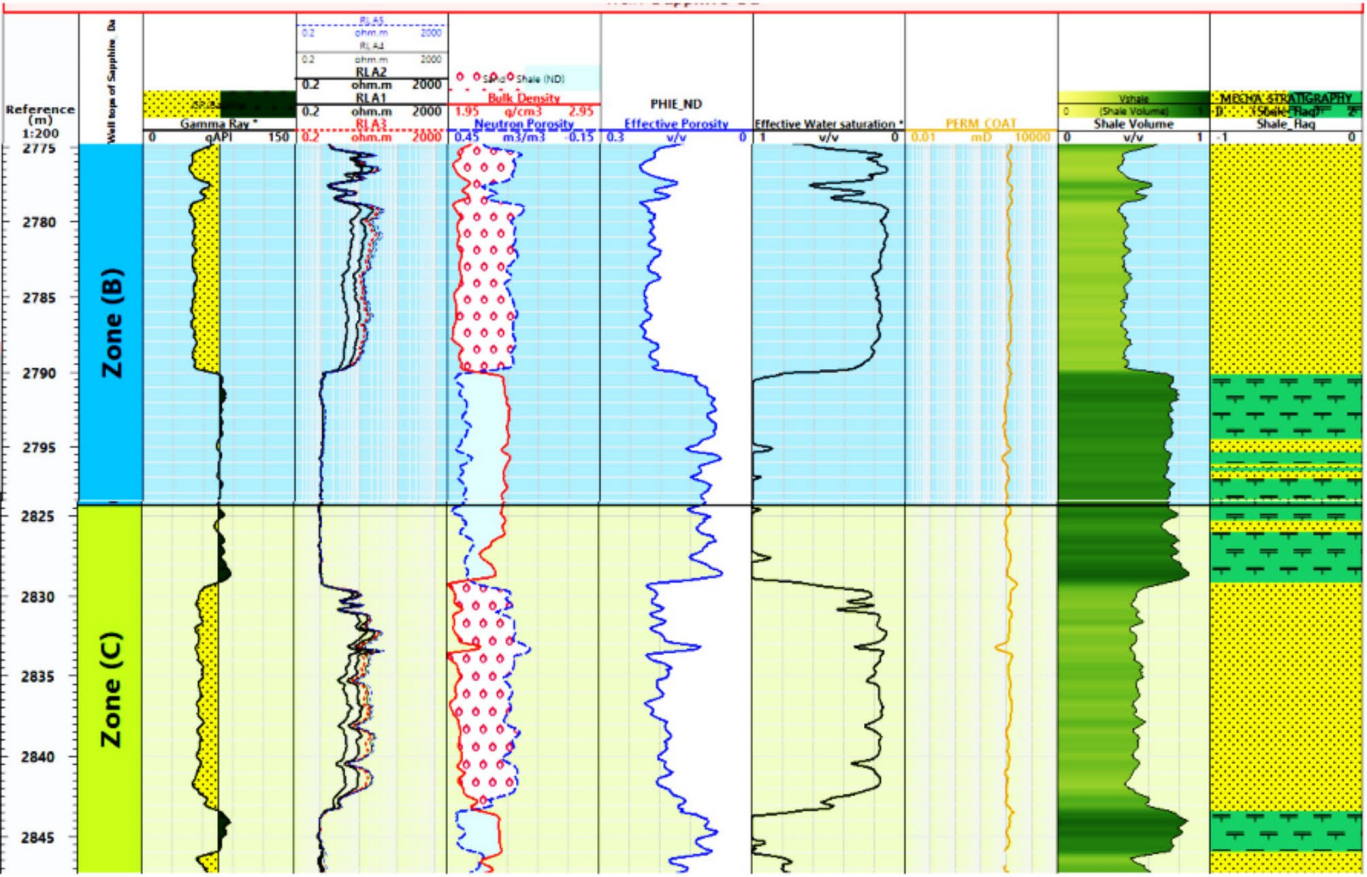
Petrophysical Interpretation log of Sapphire-Da well.

**Fig. 8:**
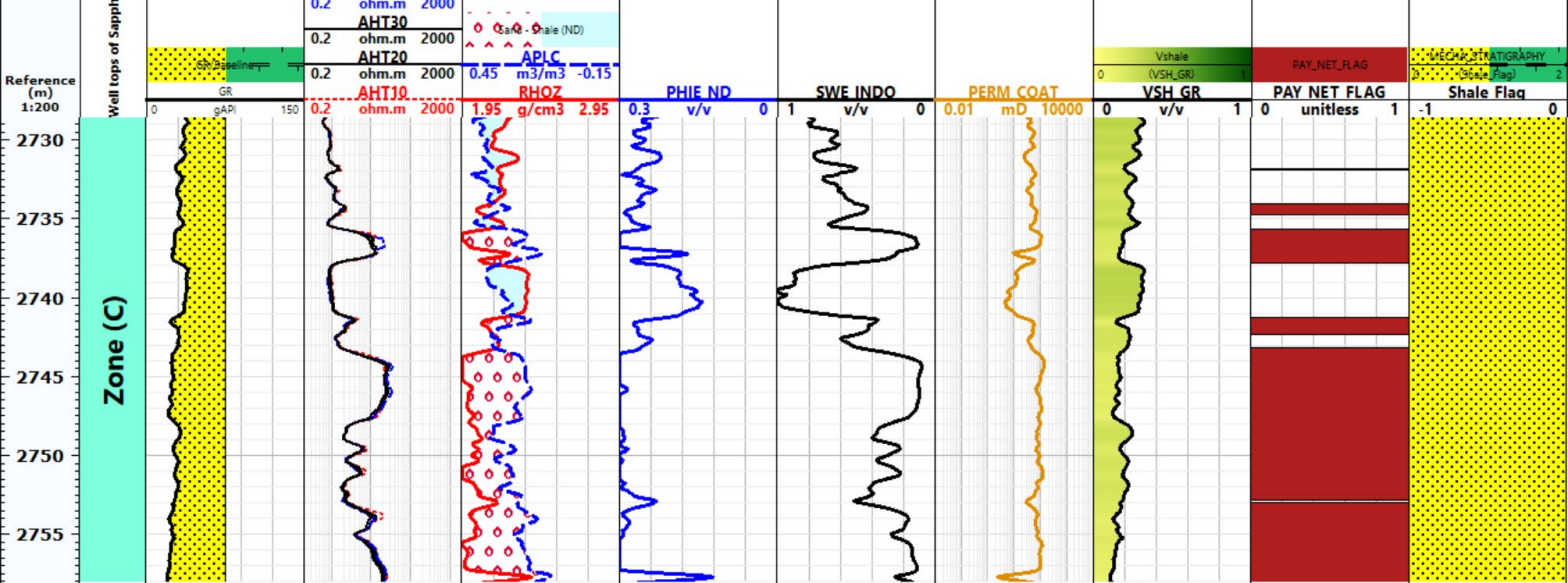
Petrophysical Interpretation log of Sapphire-Deep1 well.

**Fig. 9:**
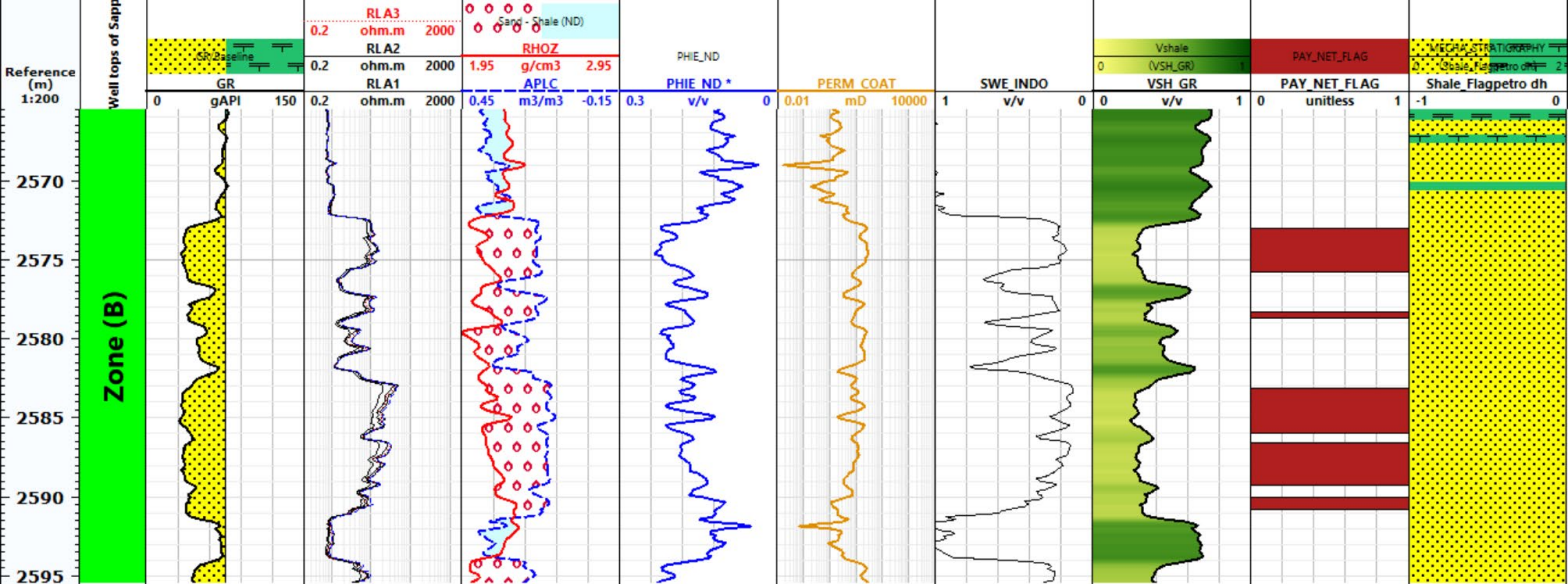
Petrophysical Interpretation log of Sapphire-Dh well.

Lithology Analysis: The lithological composition of the Sapphire-1 well is derived from gamma-ray log data. It reveals shale and sandstone formations within the reservoir, as shown in Figs. 6, 7, 8 and 9. Porosity Distribution: The average porosity in the Sapphire field is 0.18. Porosity values range from 0.03 to 0.33, reflecting diverse pore spaces within the reservoir rock. Permeability Variation: The average permeability in the Sapphire-1 well is 15.09 millidarcies. Permeability values range from 0.01 to 117.78 millidarcies, indicating heterogeneity in fluid flow properties. Reservoir Classification: Effective water saturation is a key determinant for reservoir classification. Values less than 50% classify the zone as a reservoir, while values above 50% indicate non-reservoir zones. Shale Volume Influence: The shale volume plays a critical role in reservoir classification. Shale volumes less than 35% indicate reservoir zones, while values above 35% suggest non-reservoir areas. In summary, lithological analysis using gamma-ray logs helps determine porosity, permeability, saturation, and shale volume. These findings aid in reservoir characterization and hydrocarbon recovery strategies.

#### Porosity (Φ)

The effective porosity results from the neutron log for each well (Sapphire-1, Sapphire-Da, Sapphire-Deep-1, and Sapphire-Dh) are shown in Tables 1, 2, 3 and 4.

##### Effective porosity (Φeff)

**Fig. 10:**
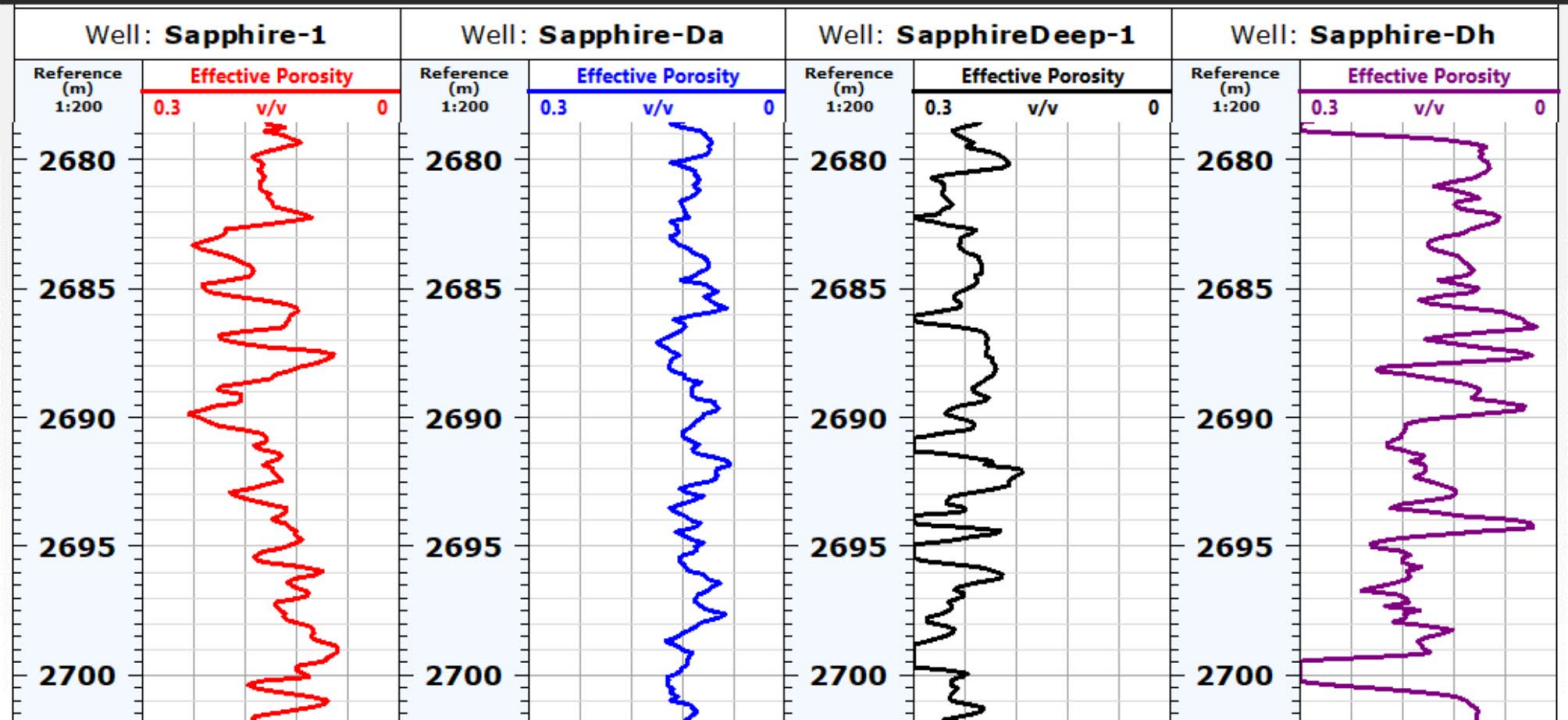
Effective porosity of sapphire wells (Sapphire-1-Sapphire-Da- Sapphire-Deep1 - Sapphire-DH).

**Fig. 11:**
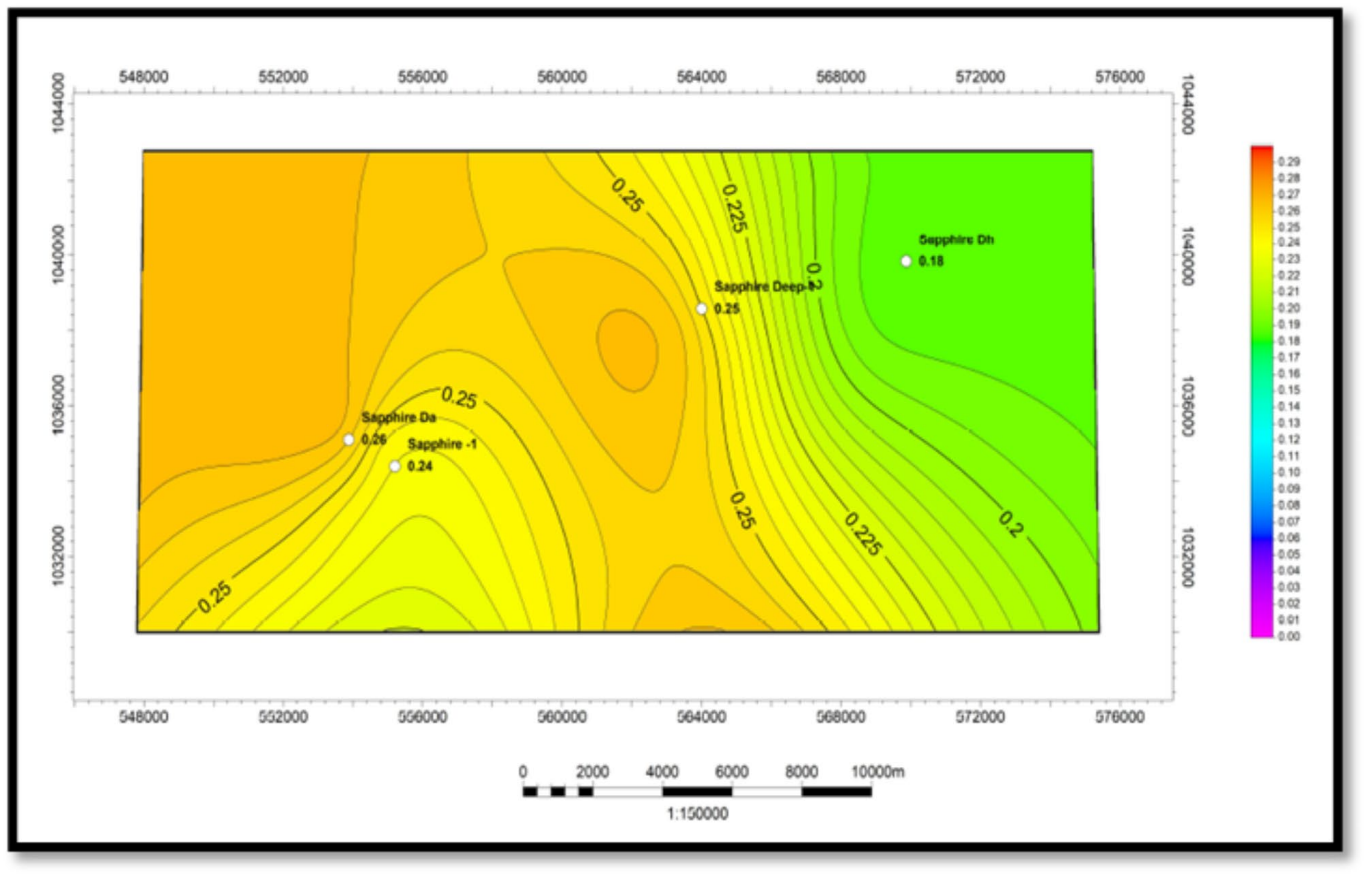
Effective porosity variation map for Kafr ElSheikh formation sapphire wells (Sapphire-1-Sapphire-Da- Sapphire-Deep1 - Sapphire-DH).

Based on the interpretation of neutron-density logs (Fig. 10), an effective porosity map was created for the Kafr ElSheikh Formation (Fig. 11). The minimum effective porosity value is approximately 14% near the Sapphire-Da well. The maximum value is around 34% near the same well. The northwest and southeast areas of the study area show promising potential for effective porosity. These areas represent the new development trends for the Kafr ElSheikh Formation.

#### Volume of shale (Vsh)

**Fig. 12:**
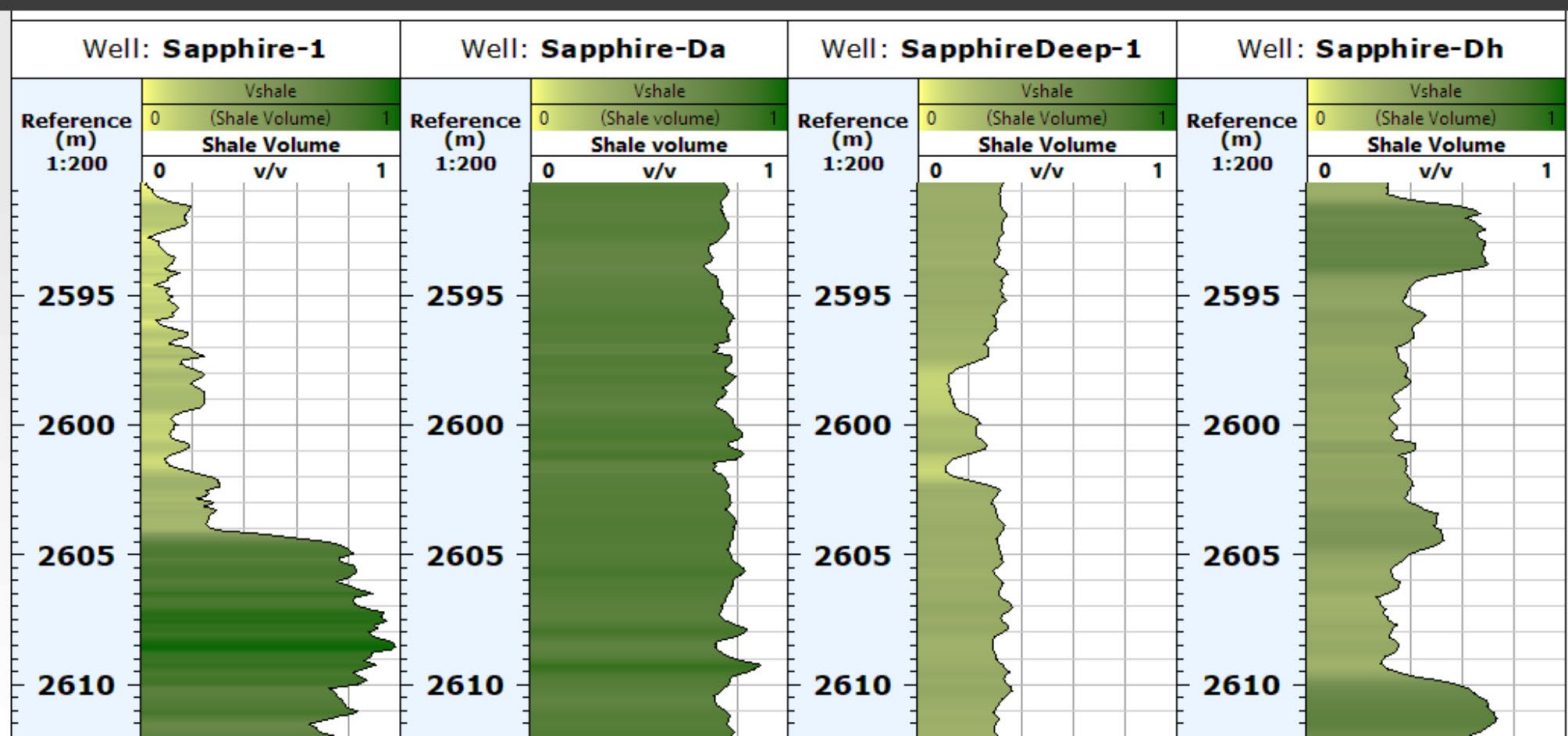
Shale contant of sapphire wells (Sapphire-1-Sapphire-Da- Sapphire-Deep1 - Sapphire-DH).

**Fig. 13:**
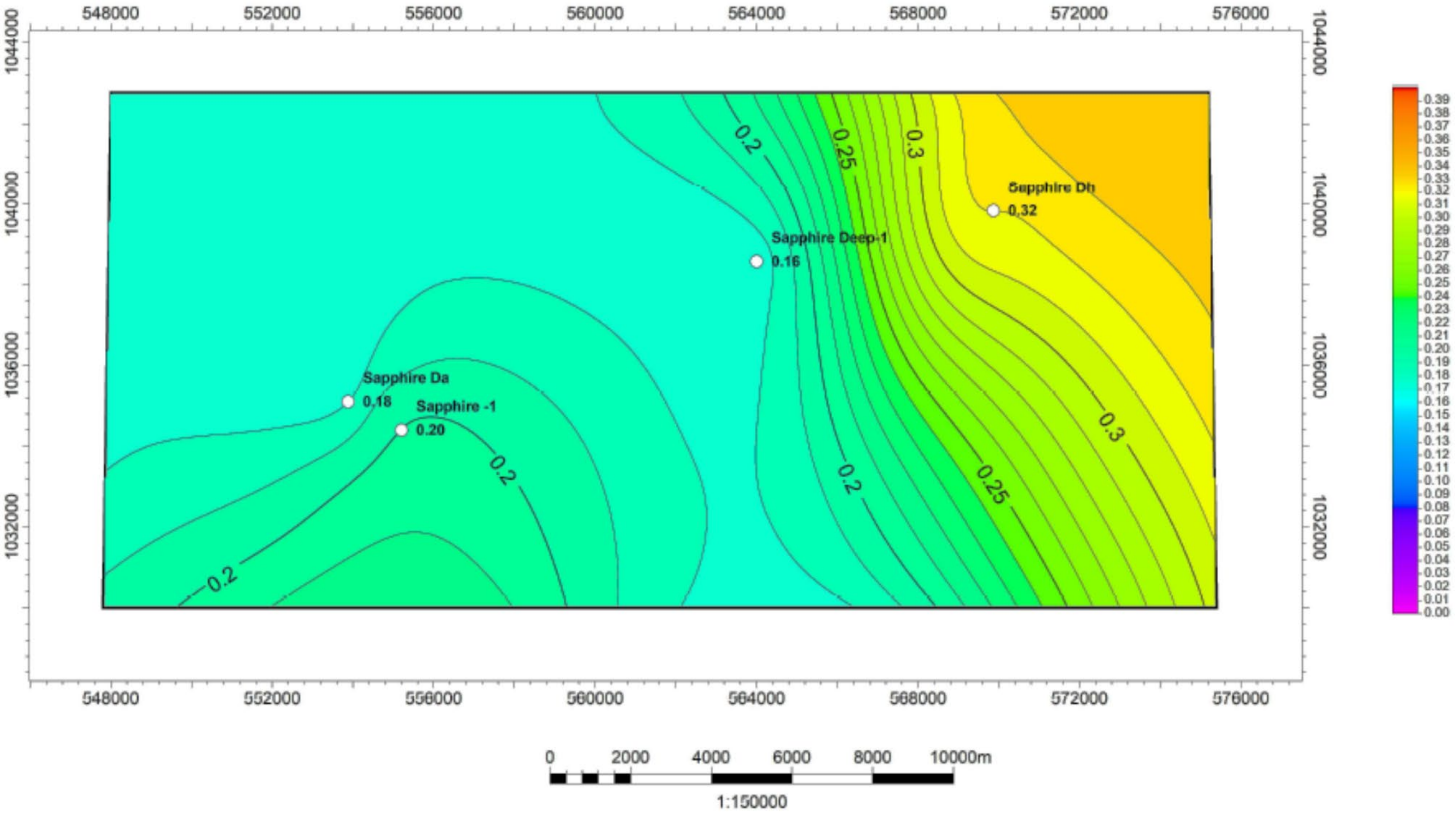
Volume of shale variation map for Kafr ElSheikh formation formation sapphire wells (Sapphire-1-Sapphire-Da- Sapphire-Deep1 - Sapphire-DH).

In order to generate a map of shale volume, the gamma ray and neutron density methods are combined to accurately calculate the amount of shale present. This approach is demonstrated in Tables 1, 2, 3 and 4 and in the interpretation logs of sapphire wells, which depict shale volume using the GR log (Fig. 12). Analysis of the resulting data for the Kafr ElSheikh Formation (Fig. 13) shows that the shale volume ranges from approximately 4–34%, with the minimum and maximum values, respectively. Furthermore, the new development of the Kafr ElSheikh Formation in the studied field is oriented towards the northwest and reveals a decrease in shale volume.

#### Water saturation (Sw)

**Fig. 14:**
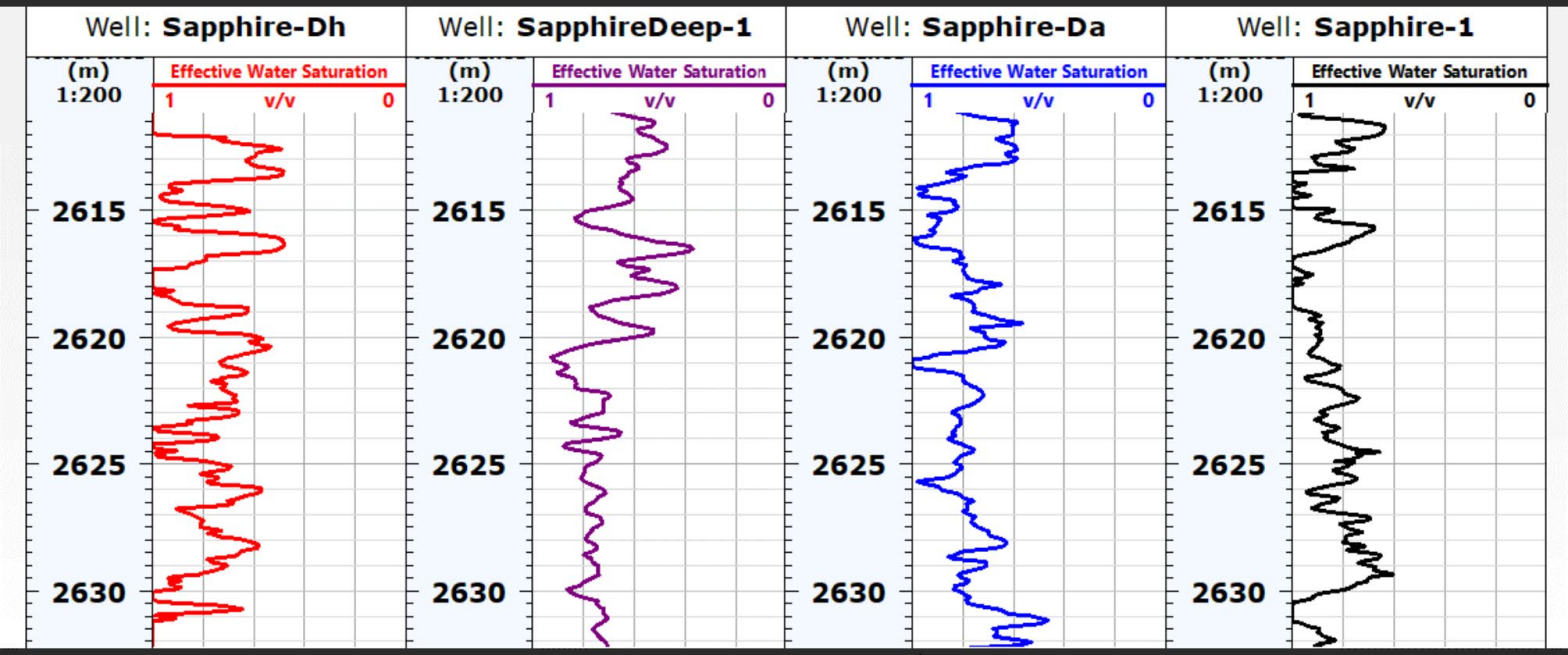
Effective Water Saturation of sapphire wells (Sapphire-1-Sapphire-Da- Sapphire-Deep1 - Sapphire-DH).

**Fig. 15:**
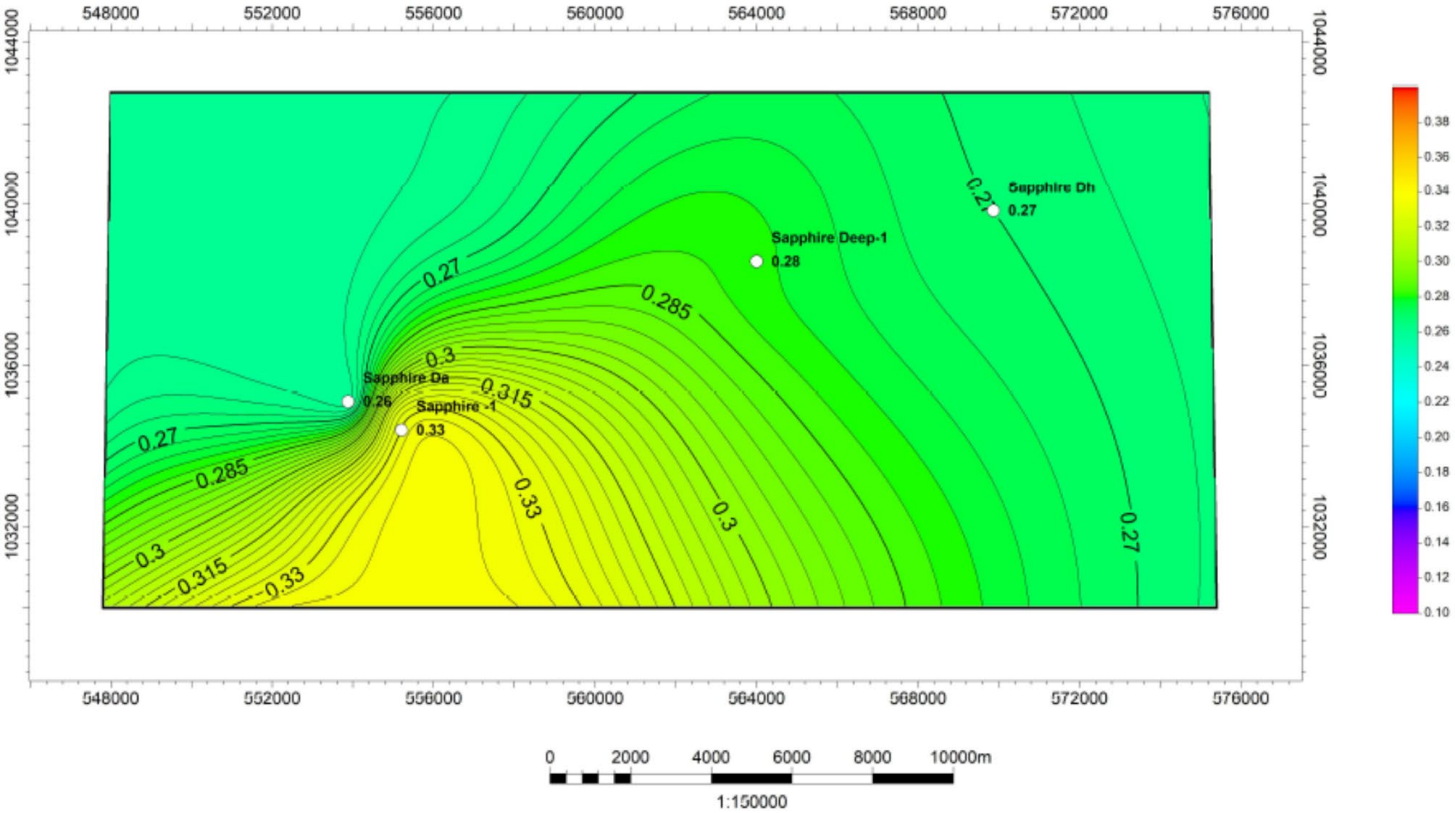
Water saturation variation map for Kafr ElSheikh formation formation sapphire wells (Sapphire-1-Sapphire-Da- Sapphire-Deep1 - Sapphire-DH).

Two methods have been employed to calculate water saturation in various wells, and the results have been presented in Tables 1, 2, 3 and 4. The Archie and Indonesia methods (effective water saturation) were used to determine which approach is superior. The interpretation logs of sapphire wells demonstrating water saturation using both the Archie and Indonesia models (Fig. 14) and the iso-effective water saturation map for the Kafr ElSheikh Formation, which was generated using both models, can be utilised to depend on the water saturation calculated using the Indonesia equation ($\:{S}_{Windo}$). The variation in water saturation for the Kafr El Sheikh Formation is depicted in a map (Fig. 15), which shows a distribution of water saturation ($\:{S}_{w}$) ranging from 15 to 45%. In the studied field, the new development for the Kafr El Sheikh Formation is oriented southeast-northwest and exhibits less water saturation in the Kafr El Sheikh Formation.

#### Gas saturation (Sg)

**Fig. 16:**
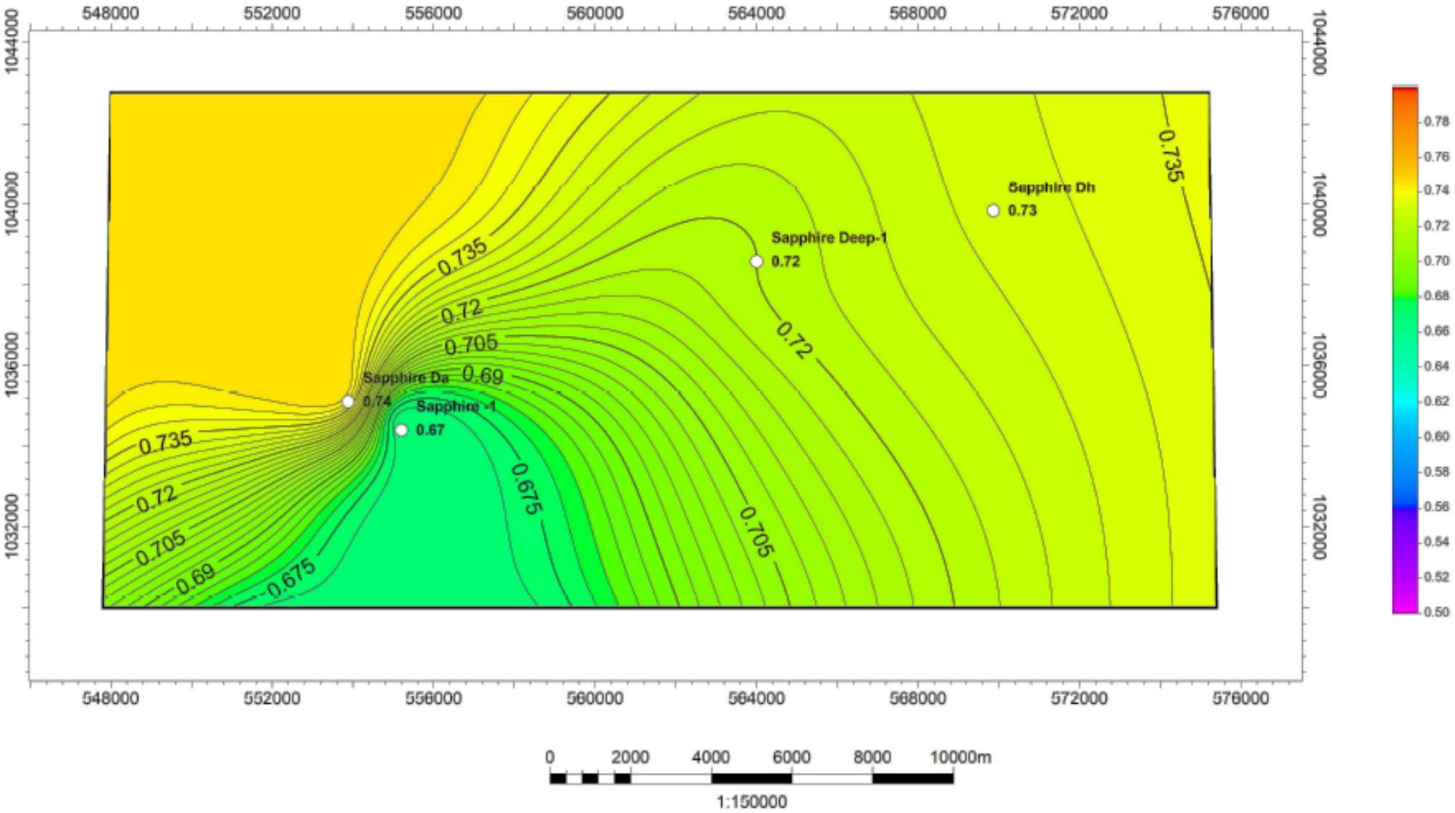
Gas saturation variation map for Kafr ElSheikh formation formation sapphire wells (Sapphire-1-Sapphire-Da- Sapphire-Deep1 - Sapphire-DH).

Gas saturation for the Kafr El Sheikh Formation is shown in Tables 1, 2, 3 and 4. An iso-hydrocarbon saturation map (Fig. 16) reveals that gas saturation ranges from 55 to 85%. The new development trends in the field point towards the northwest-southwest, suggesting good potential for reservoir functionality.

#### Permeability (k)

**Fig. 17:**
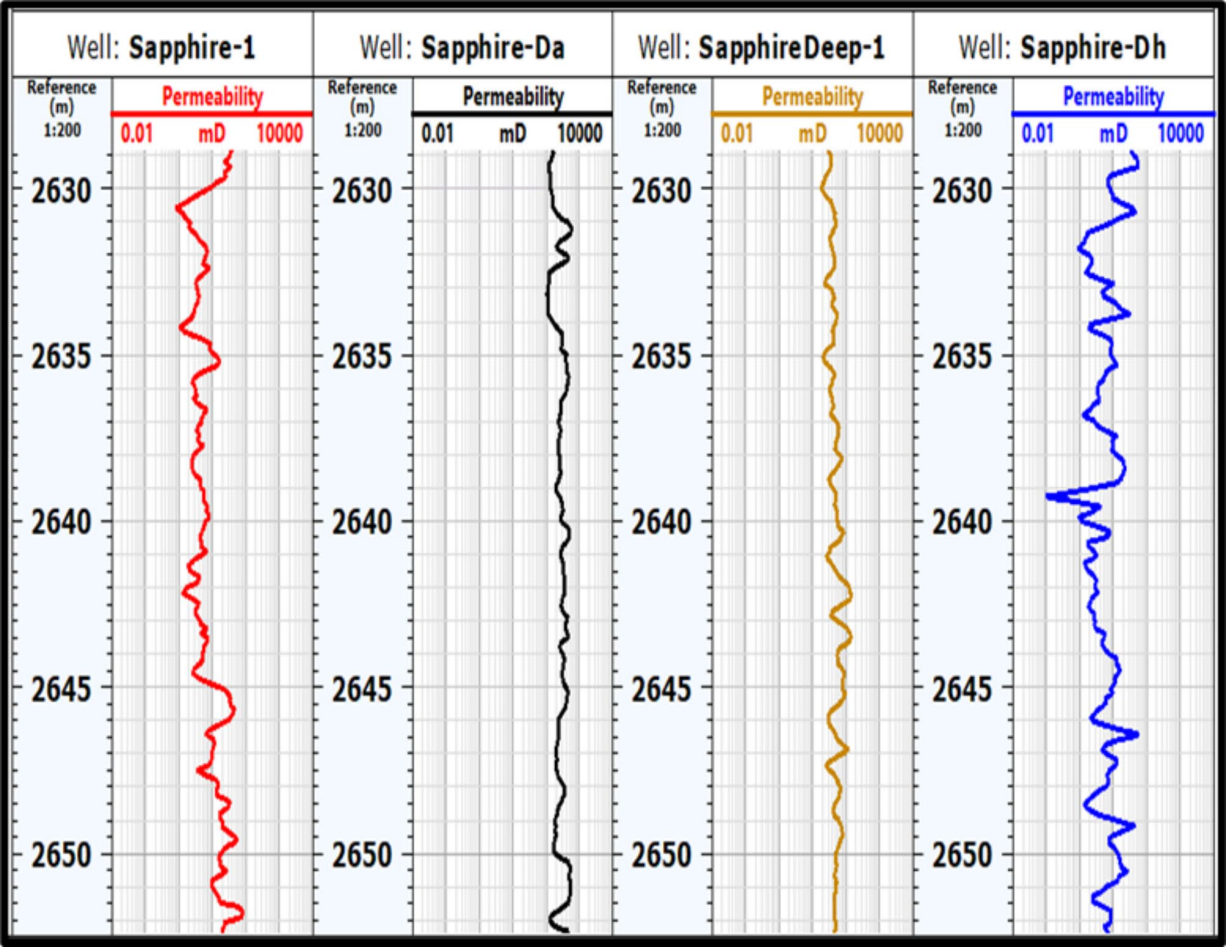
Permeability of sapphire wells (Sapphire-1-Sapphire-Da- Sapphire-Deep1 - Sapphire-DH).

**Fig. 18:**
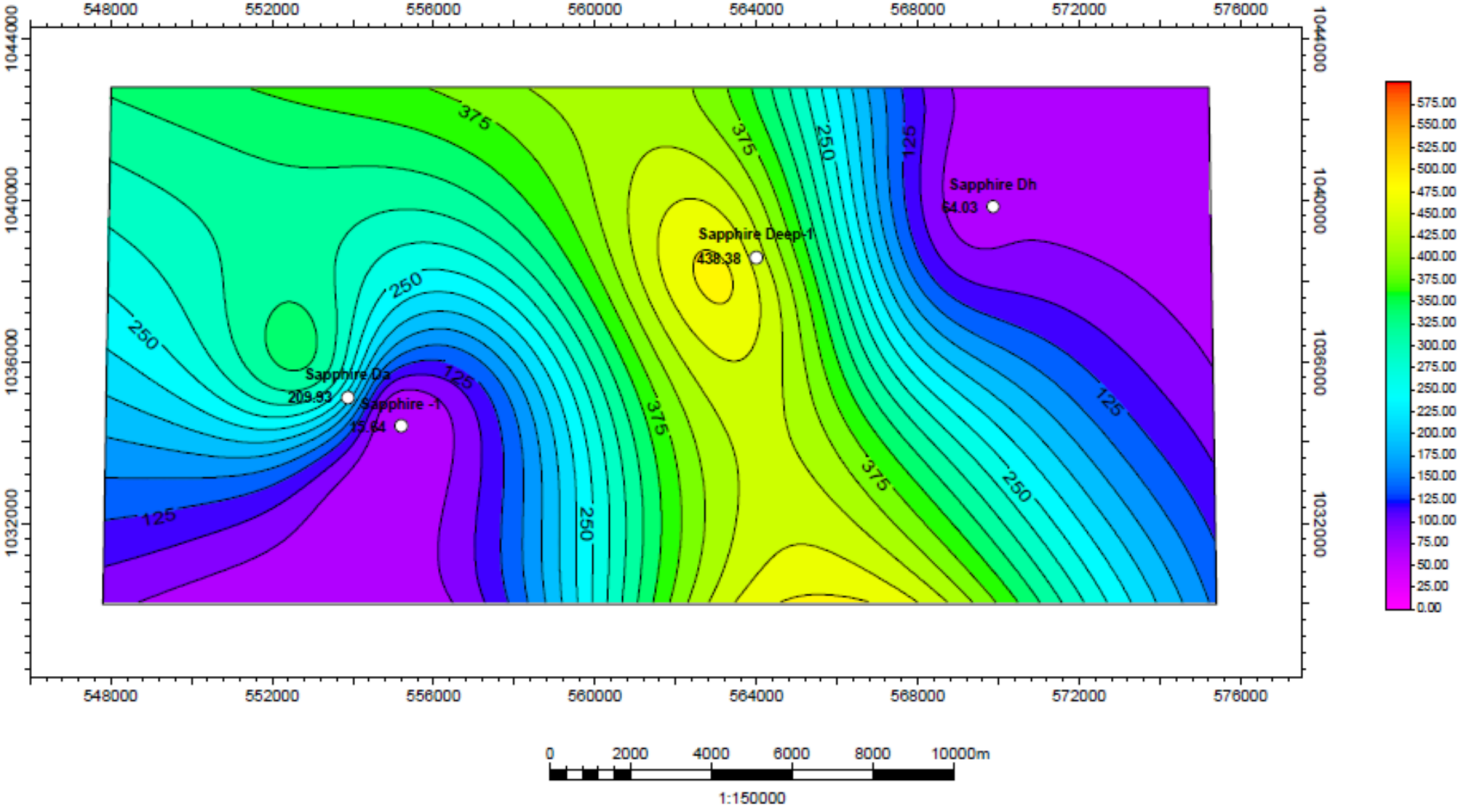
Permeability variation map for Kafr ElSheikh formation formation sapphire wells (Sapphire-1-Sapphire-Da- Sapphire-Deep1 - Sapphire-DH).

Permeability in the Kafr El Sheikh Formation was determined using interpretation logs of effective porosity (Fig. 17). A permeability map for the formation (Fig. 18) shows a range of values from 5.57 mD to 438.38 mD. New developments in the northwest and southeast areas indicate a high potential for effective porosity and storage capacity.

#### Netpay thickness

**Fig. 19:**
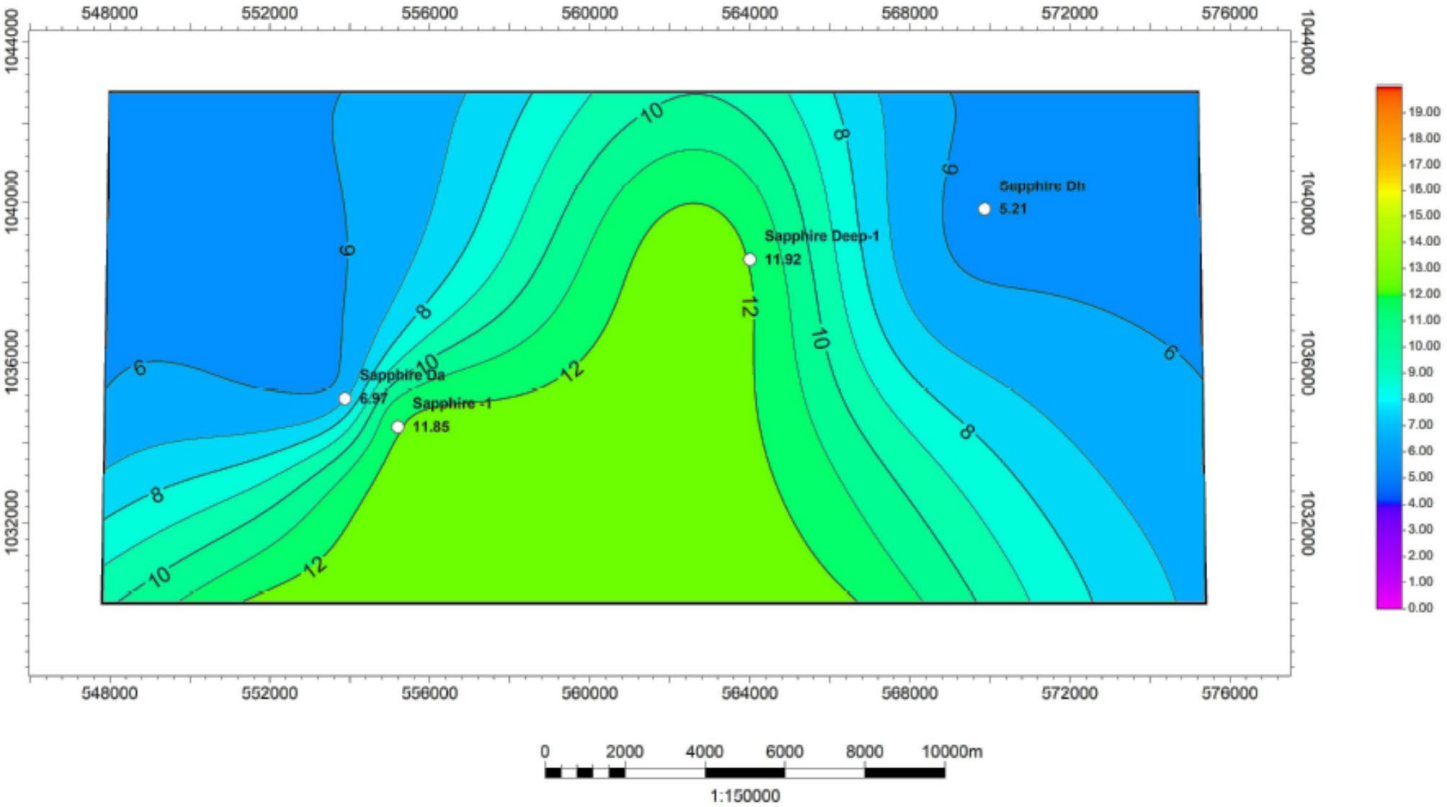
Netpay thickness variation map for Kafr ElSheikh formation formation sapphire wells (Sapphire-1-Sapphire-Da- Sapphire-Deep1 –SapphireDH).

Net Pay Thickness (Tables 1, 2, 3 and 4) is calculated for each zone in the Kafr ElSheikh Formation using data from various wells. These results are used to create a net pay thickness map for the formation, shown in Fig. 19. The map shows thickness values ranging from 0.46 m to 23.8 m. In the studied field, new development in the Kafr ElSheikh Formation is oriented towards the northwest-southwest regions. These regions show significant potential for reservoir function.

### Lithological identification cross plots

**Fig. 20:**
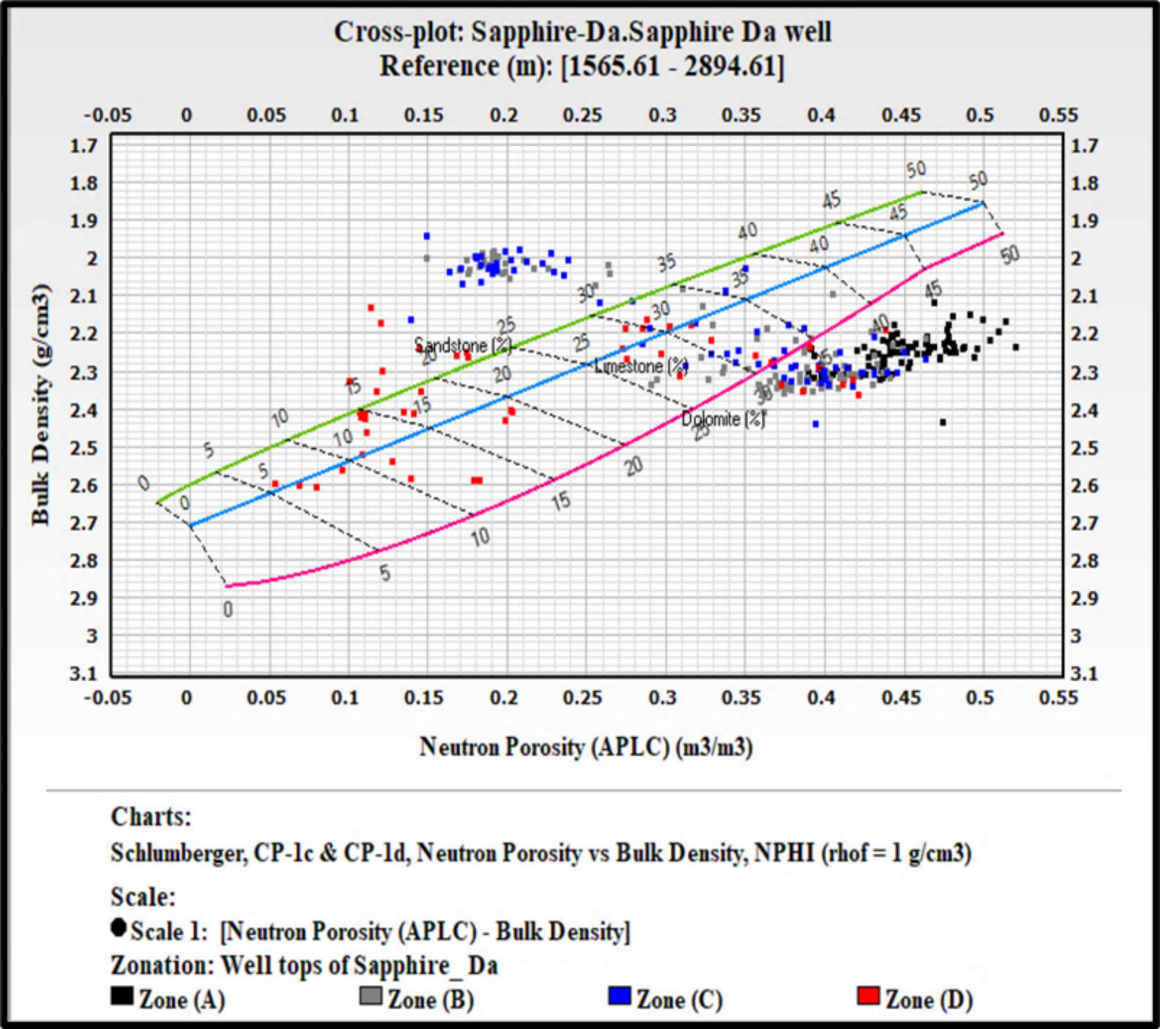
Neutron Density cross plot for Sapphire Da well.

**Fig. 21:**
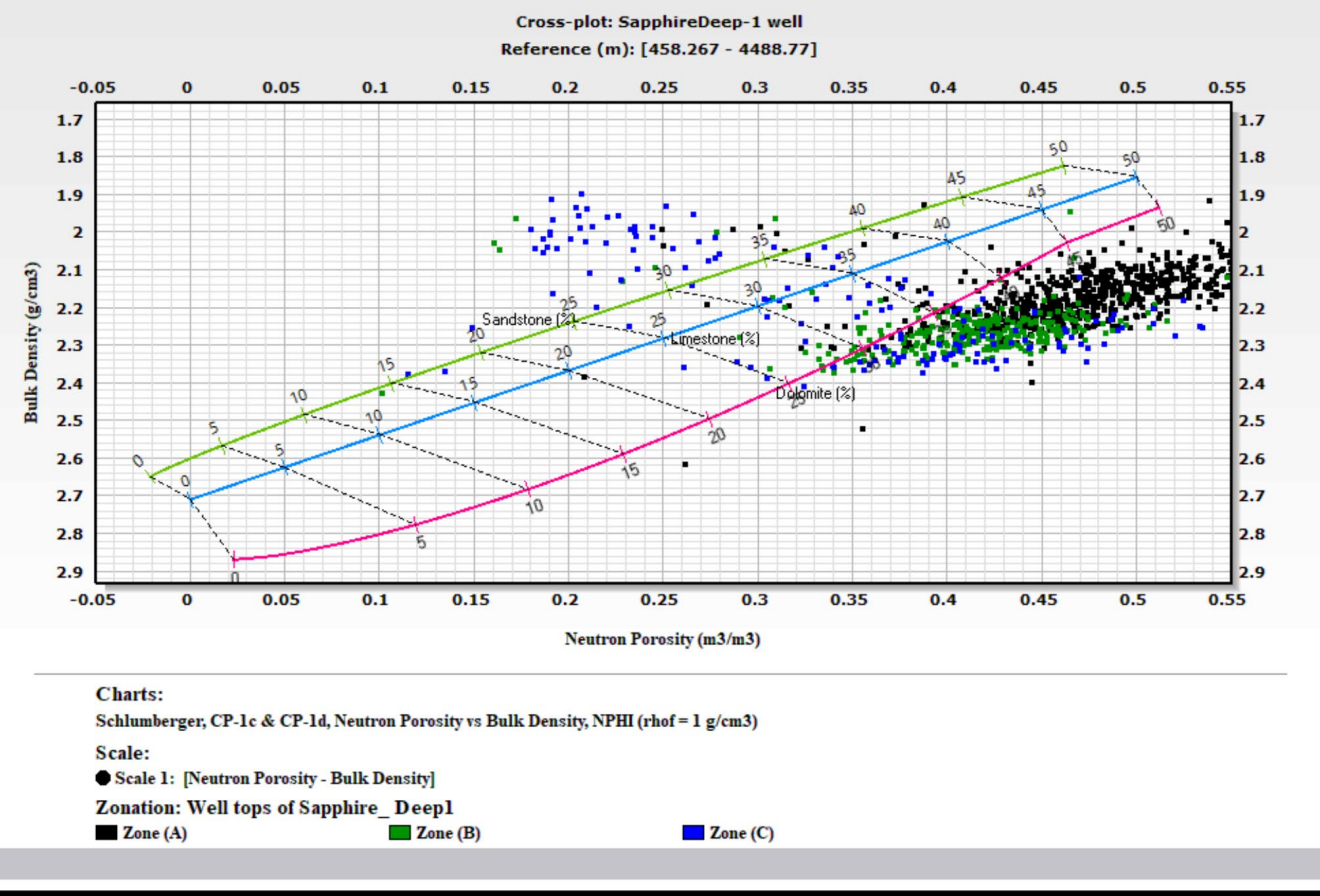
Neutron Density cross plot for Sapphire Deep1 well.

**Fig. 22:**
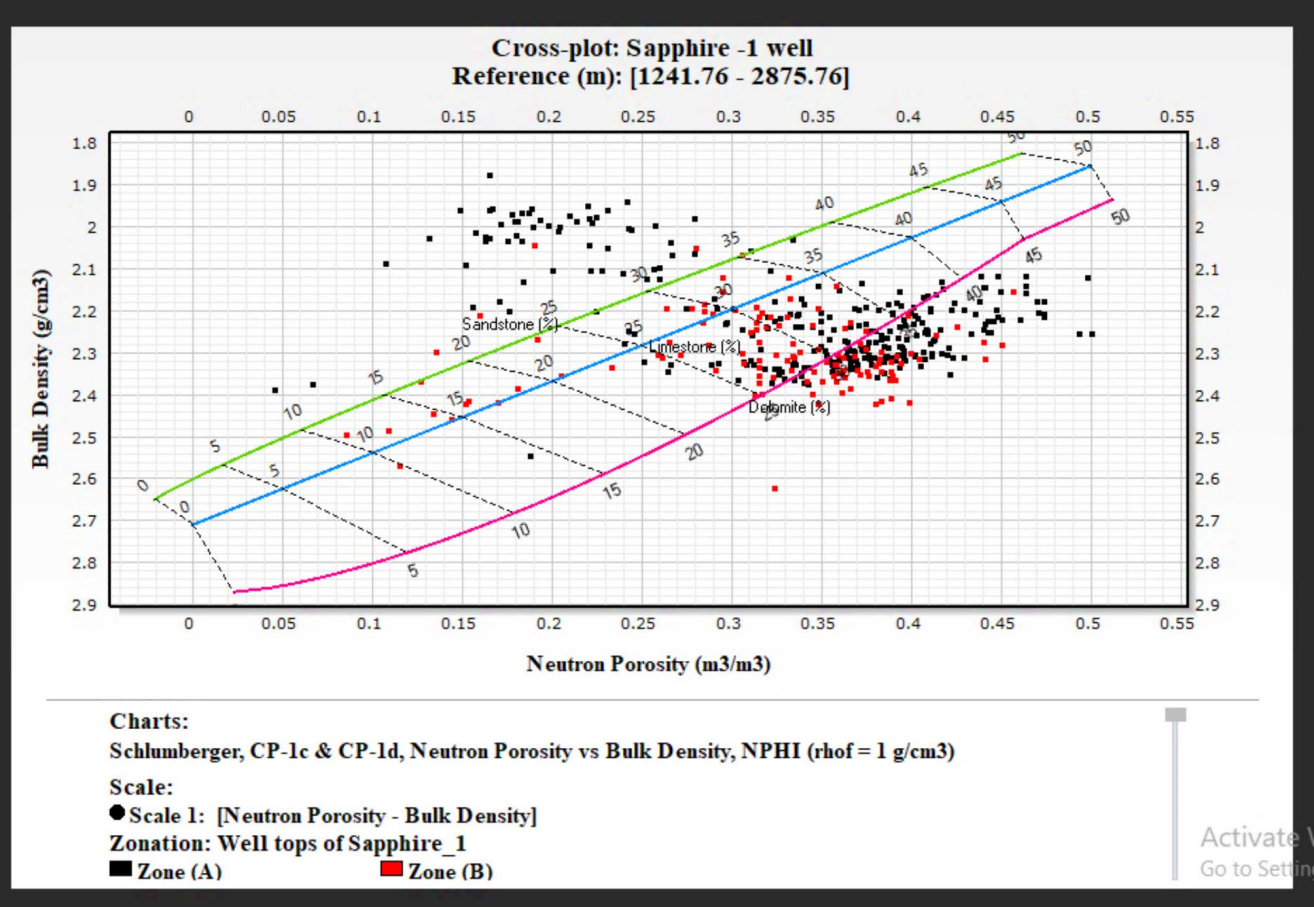
Neutron Density cross plot for Sapphire 1 well.

**Fig. 23:**
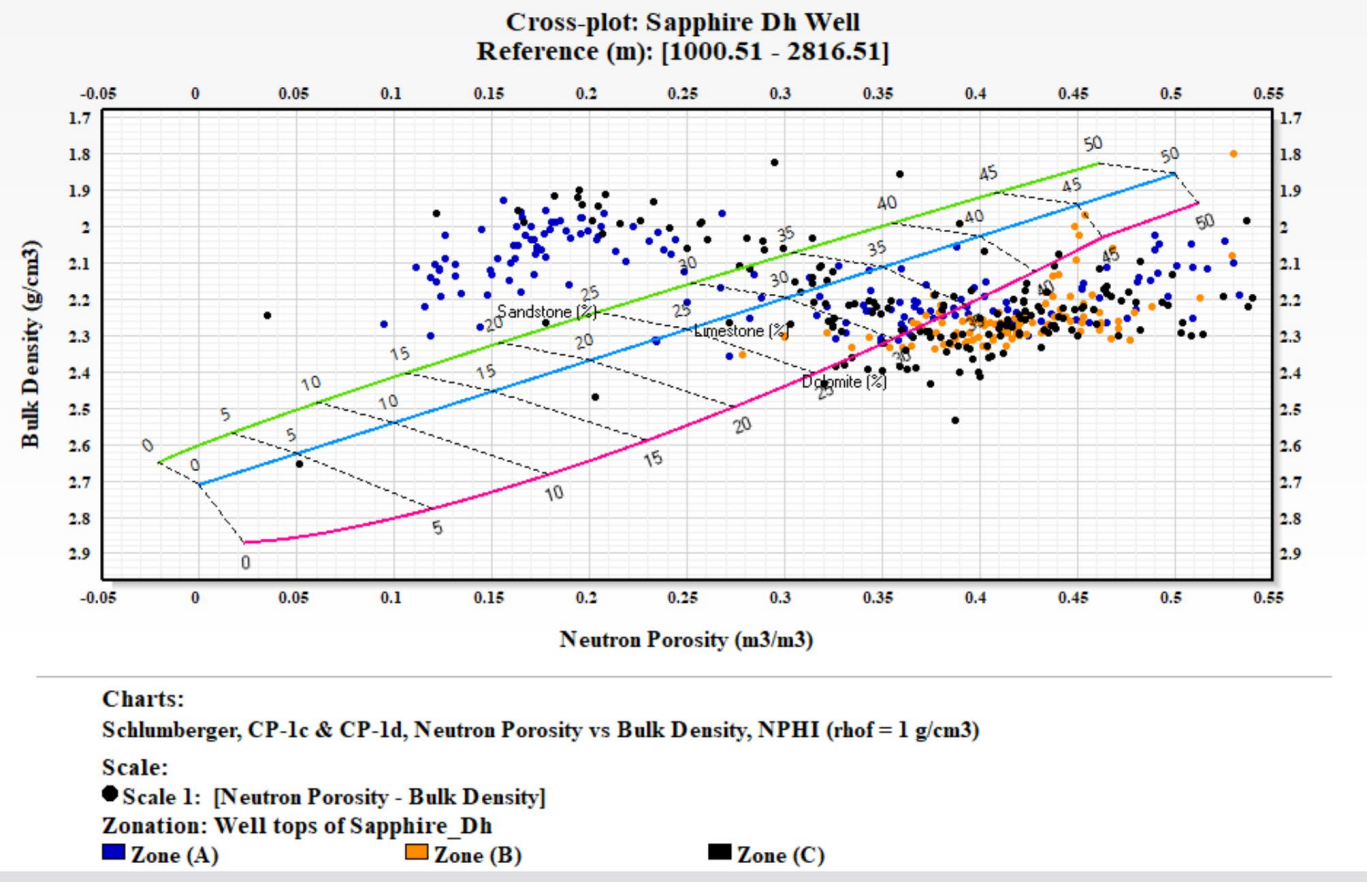
Neutron Density cross plot for Sapphire Dh well.

The precise identification of lithology within a formation is critical in the evaluation process. Well logs are invaluable tools in this endeavor. Among the various log types, density, neutron, sonic, and gamma-ray logs are the most effective for lithological identification. This study focuses on the Kafr ElSheikh Formation in multiple wells, using a neutron-density cross-plot for lithological identification. This study builds on methodologies discussed by Poupon et al. (1970)^10,12^, . These researchers explored cross-plot combinations for composite logs analysis. This study uses the neutron-density cross-plot method to interpret the lithological composition of the Kafr ElSheikh Formation. The method aligns with the tradition of using cross-plots to understand subsurface formations. It enhances the reliability of lithological identification in the studied geological context. Additionally, a visual aid based on gamma-ray logs clarifies lithology. The figure shows that the lithology is a mix of shale and sandstone. The graphical representation helps understand lithological variations within the Kafr ElSheikh Formation. It contributes to a more detailed evaluation of the reservoir. Figure (20) illustrates the Sapphire Da well. A substantial portion of data points is positioned between the limestone and sandstone lines. This indicates the coexistence of shale and sandstone lithologies, with porosity ranging from 14 to 34%. Scattered points between the limestone and dolomite lines suggest mixed lithology. This finding emphasizes the complex geological nature of the Sapphire Da well. It highlights the importance of accurate lithological identification for a comprehensive understanding of the reservoir. Figure (21) shows the analysis of Sapphire Deep 1. The plotted locations mainly align with the sandstone line. The average porosity ranges from 23 to 30%. Some points between the limestone and sandstone lines indicate a blend of sandstone and shale. This reveals the complex geology of Sapphire Deep 1. In the analysis of Sapphire 1 (Fig. 22), a predominant cluster aligns with the sandstone line. The average porosity ranges from 20 to 27%. Points between the limestone and sandstone lines indicate mixed lithology. This finding enhances understanding of the reservoir’s petrophysical dynamics. In Sapphire Dh well(Fig. 23), the neutron-density cross-plot shows that most points are around the sandstone line. The average porosity ranges from 15 to 22%. Scattered points between the limestone and dolomite lines suggest mixed lithology. This could result from the shale effect or borehole conditions.

### Cutoff determination

#### Cutoff of shale content

**Fig. 24:**
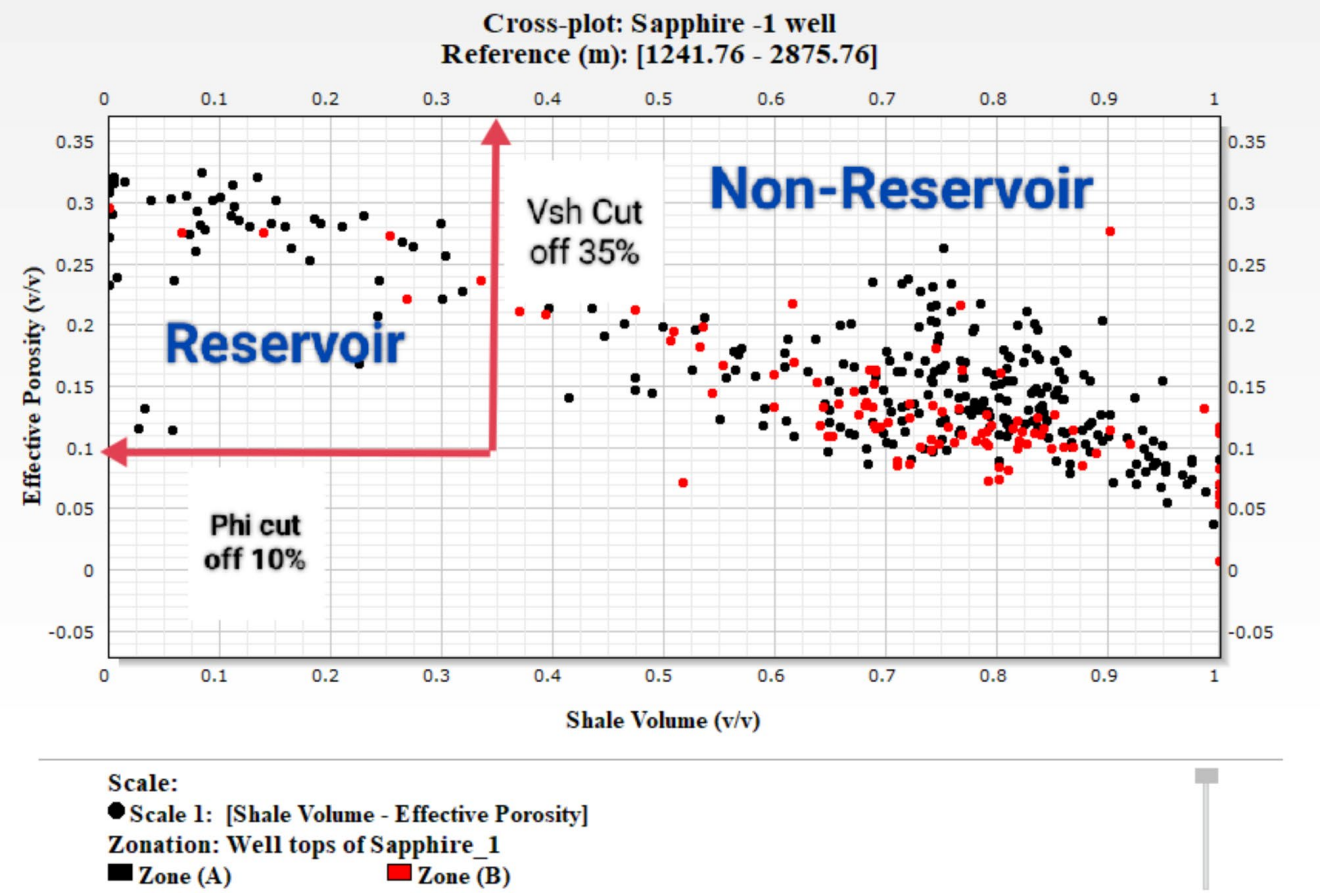
Cut-off determination is based on a cross plot of shale content vs. porosity and a gamma ray log of Sapphire − 1 well.

The cutoff value for shale content has been determined using the shale content-effective porosity relationship of multiple wells and gamma ray logs. This cutoff value, shown in (Fig. 24) as a cross plot of gamma rays and shale content-porosity, is 0.35. Rocks with a shale content less than or equal to 35% are classified as reservoir rocks, while those with a shale content greater than 35% are classified as non-reservoir rocks. This approach is commonly used to identify sand intervals and differentiate them from shale. (Ghanima et al., 2020^13^).

#### Cut-off of water saturation

**Fig. 25:**
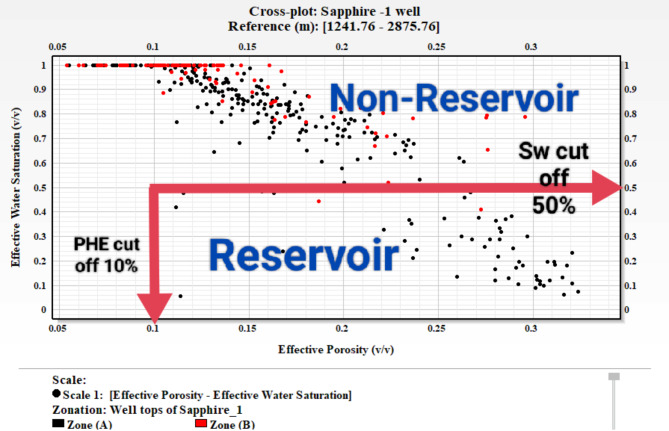
Cut-off determination is based on a cross plot of shale content vs. porosity and a gamma ray log of Sapphire − 1 well.

The effective water saturation-effective porosity cross plot and gamma ray log are frequently utilized to differentiate productive (pay) intervals from non-productive (non-pay) intervals in porous formations (Fig. 25). In this instance, a water saturation value of less than 50% is used as the cutoff for identifying pay zones, signifying that interval with water saturation greater than 50% are classified as non-productive. This cutoff is based on the presumption that oil or gas is improbable to be present in intervals with high water saturation values. However, the effectiveness of this cutoff may fluctuate depending on the specific geological conditions of the reservoir under study.

#### Porosity cut-off

**Fig. 26:**
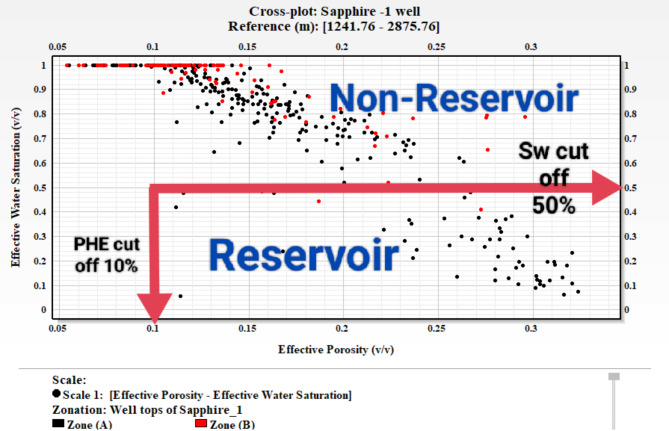
Porosity-water saturation cross plot for cut-off determination.

The gross sand interval’s porosity cutoff is an important criterion for distinguishing between different types of sand intervals. In particular, a minimum porosity value is required for the sand to be permeable enough to allow oil and gas to flow through it (Fig. 26). Porosity levels of 10% or higher are generally considered sufficient for permeability and are therefore used to distinguish between reservoirs and non-reservoirs^14^ El-Din et al., 2013. However, it’s important to note that other factors, such as the nature of the pore space, can also affect permeability and must be taken into account when evaluating a potential reservoir.

### Permeability-effective porosity relationship in the Sapphire field

The investigation into the Sapphire gas field in the Nile Delta necessitates a meticulous analysis of the intricate relationship between porosity and permeability. Porosity, denoting the void spaces within the geological formations, assumes a pivotal role in determining the reservoir’s hydrocarbon storage capacity. In the specific context of the Sapphire field, a profound comprehension of porosity is indispensable for accurate estimations of gas reserves.

Conversely, permeability delineates the rock’s capacity to allow fluid flow, governed by factors such as pore size, connectivity, and composition. In the case of the Sapphire field, permeability emerges as a critical parameter influencing the ease of gas migration through the reservoir rocks. A nuanced exploration of porosity and permeability in the Sapphire field entails a multidisciplinary approach, encompassing core analysis, well logging, and geophysical surveys. The integration of these datasets facilitates the creation of robust reservoir models, contributing significantly to reservoir management and the formulation of efficient extraction strategies.

In summary, the intricate interplay between porosity and permeability in the Sapphire gas field serves as a cornerstone in comprehending reservoir characteristics, thereby guiding exploration and production endeavors in the Nile Delta.

**Fig. 27:**
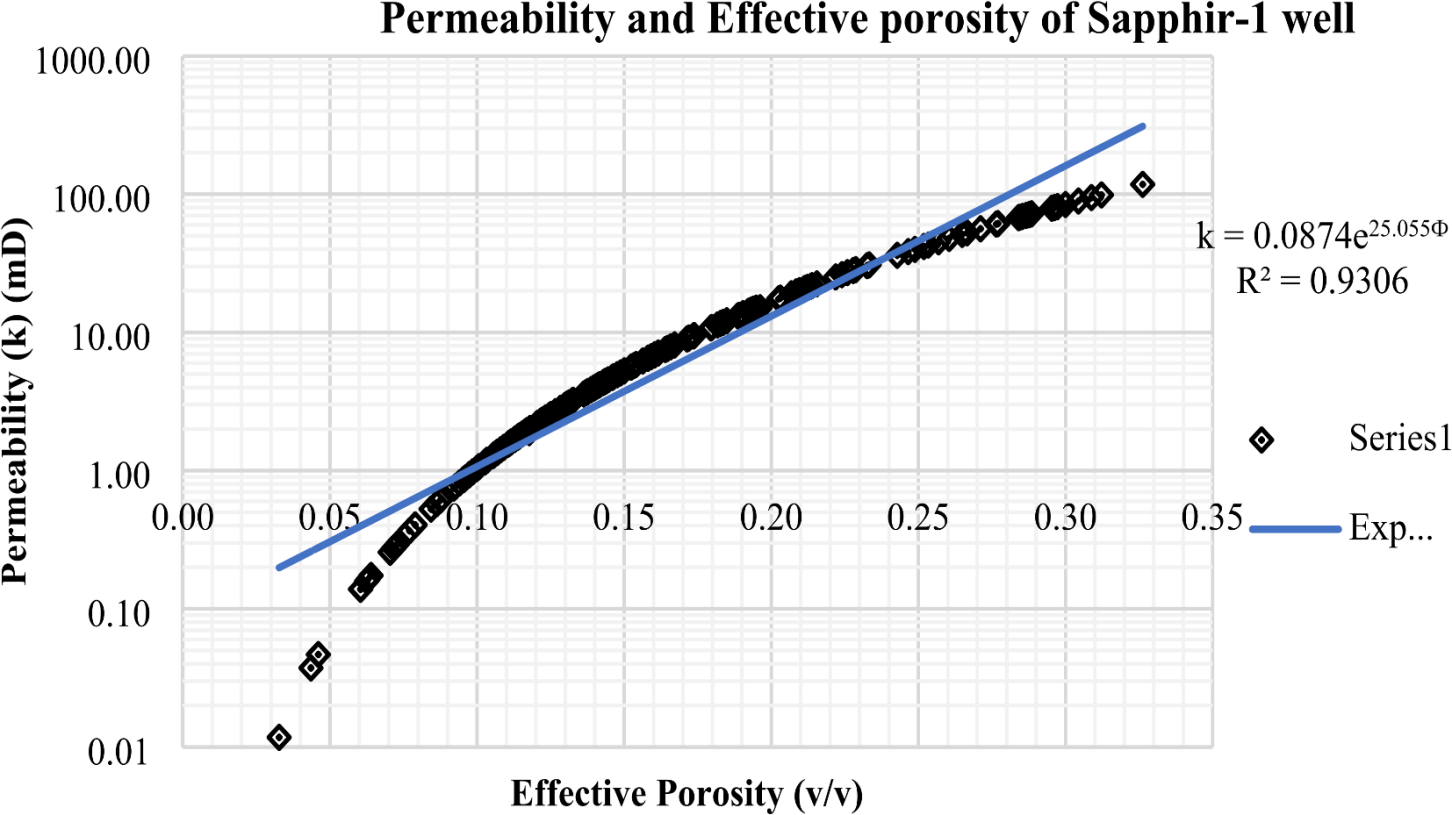
Permeability – effective porosity relationship in Sapphire − 1 well.

**Fig. 28:**
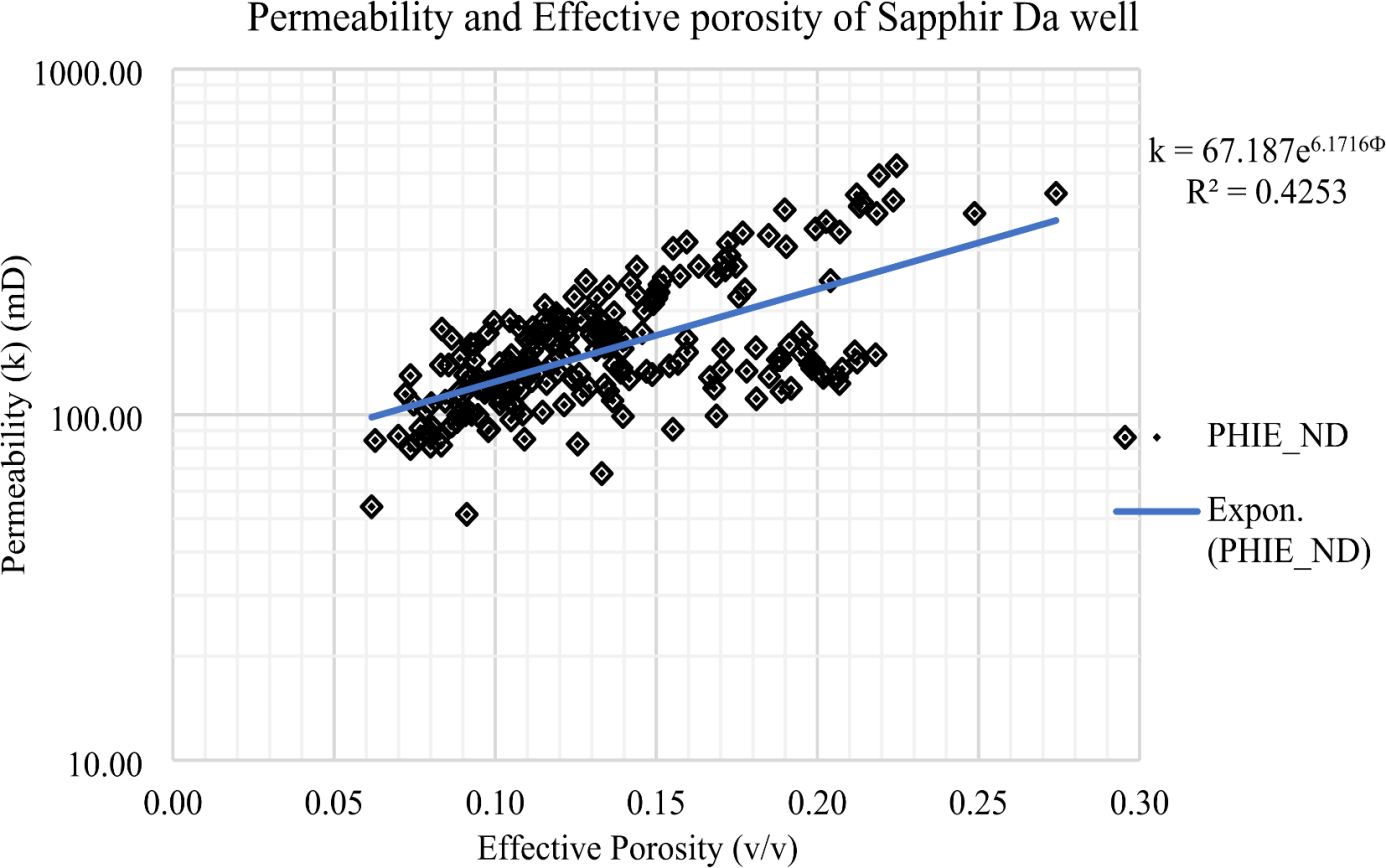
Permeability – effective porosity relationship in Sapphire -Da well.

**Fig. 29:**
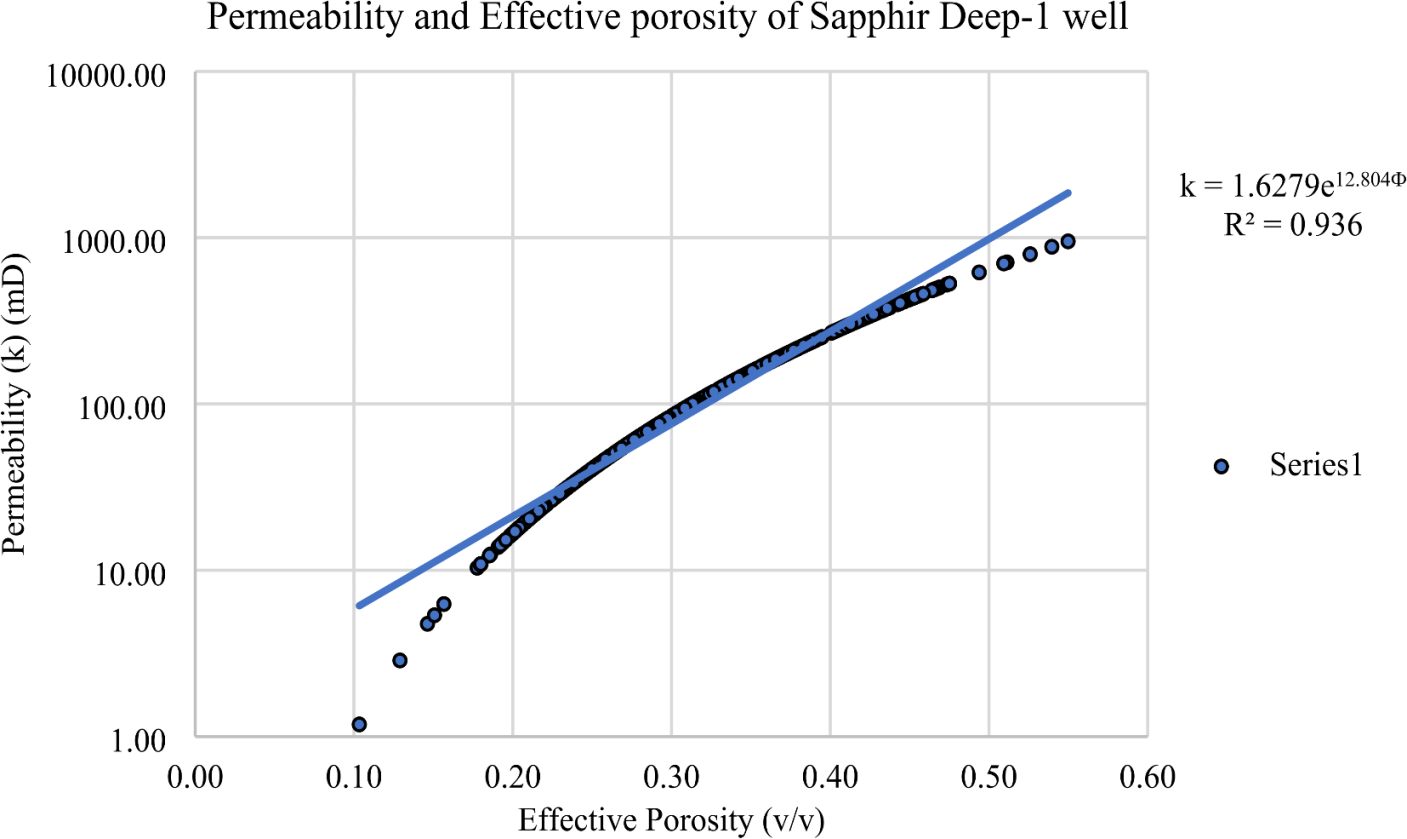
Permeability – effective porosity relationship in Sapphire -Deep1 well.

**Fig. 30:**
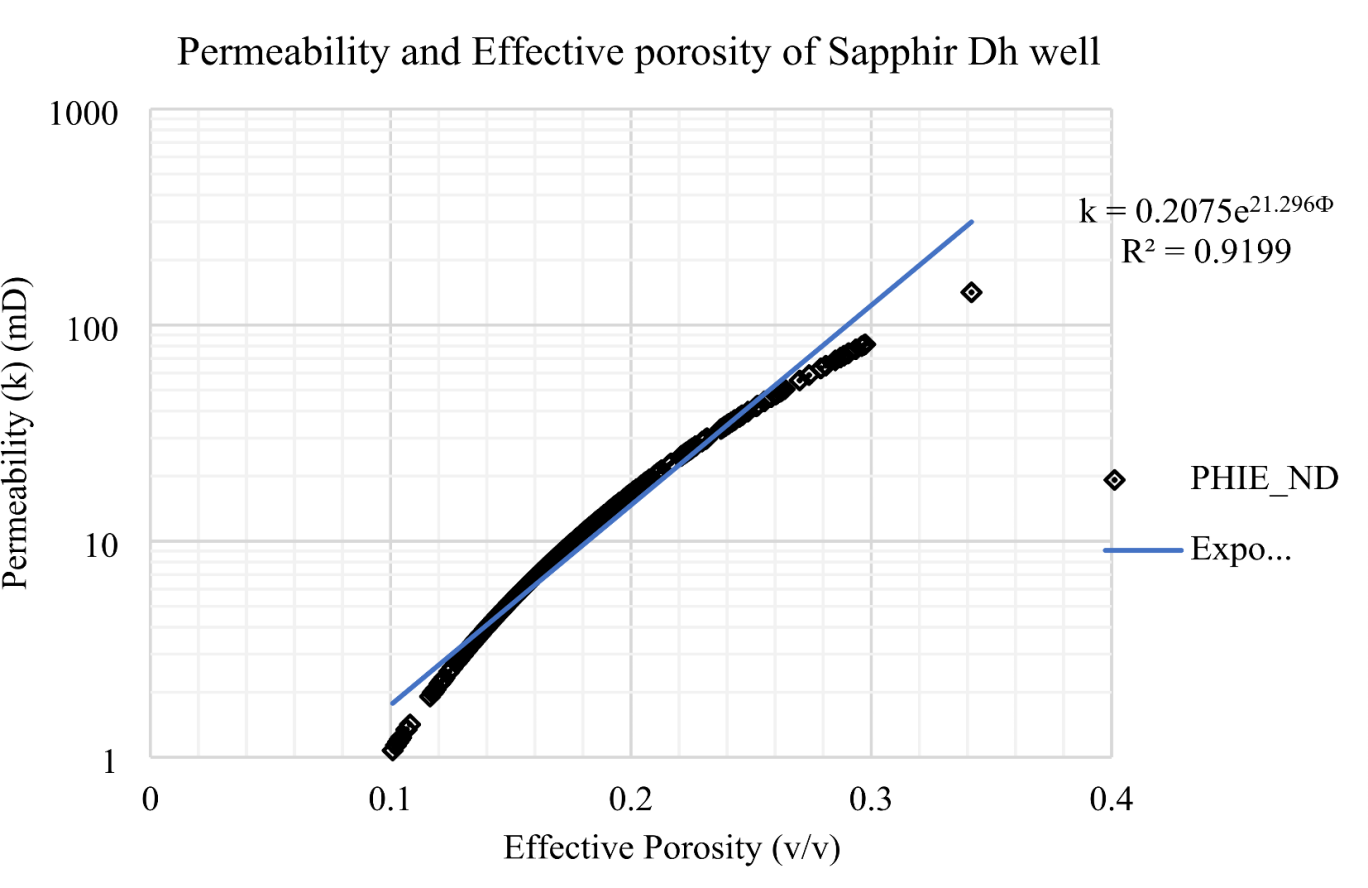
Permeability – effective porosity relationship in Sapphire -Dh well.

**Table 1:** Final table of petrophysical results of sapphire − 1 well.

Workflow Table Result MD of Sapphire-1										
Zone name		Top (m)	Bottom (m)	Gross (m)	Net (m)	*N*/G	$\:{\text{V}}_{\text{s}\text{h}}$(%)	$\:{{\Phi\:}}_{eff}\:$(%)	$\:{\text{S}}_{W}$(%)	$\:{\text{S}}_{g}$(%)
1	Zone A	2660	2800	140	23.80	0.17	0.11	0.28	0.22	0.78
2	Zone B	2800	2860	60	0.60	0.01	0.29	0.26	0.41	0.59

**Table 2:** Final table of petrophysical results of Sapphire -Da well.

Workflow Table Result MD of Sapphire-Da										
Zone name		Top (m)	Bottom (m)	Gross (m)	Net (m)	*N*/G	$\:{\text{V}}_{\text{s}\text{h}}$(%)	$\:{{\Phi\:}}_{eff}\:$(%)	$\:{\text{S}}_{W}$(%)	$\:{\text{S}}_{g}$(%)
1	Zone A	2621	2667	46	0.46	0.01	33	34	45	55
2	Zone B	2775	2829	54	14.04	0.26	4	29	15	85
3	Zone C	2829	2859	30	12.90	0.43	25	24	18	83
4	Zone D	2859	2875	16	0.48	0.03	9	14	25	75

**Table 3:** Final table of petrophysical results of Sapphire -Deep1 well.

Workflow Table Result MD of Sapphire-Deep1										
Zone name		Top (m)	Bottom (m)	Gross (m)	Net (m)	*N*/G	$\:{\text{V}}_{\text{s}\text{h}}$ (%)	$\:{{\Phi\:}}_{eff}\:$(%)	$\:{\text{S}}_{W}$(%)	$\:{\text{S}}_{g}$(%)
1	Zone A	2206	2578	372	3.72	0.01	0.15	0.29	0.37	0.63
2	Zone B	2578	2726	148	4.44	0.03	0.17	0.27	0.27	0.73
3	Zone C	2726	2801	75	23.25	0.31	0.17	0.27	0.23	0.77

**Table 4:** Final table of petrophysical results of Sapphire -Dh well.

Workflow Table Result MD of Sapphire-Dh										
Zone name		Top (m)	Bottom (m)	Gross (m)	Net (m)	*N*/G	$\:{\text{V}}_{\text{s}\text{h}}$ (%)	$\:{{\Phi\:}}_{eff}\:$(%)	$\:{\text{S}}_{W}$(%)	$\:{\text{S}}_{g}$(%)
1	Zone A	2419	2562	143	1.43	0.01	34	28	34	66
2	Zone B	2562	2638	76	14.44	0.19	30	20	23	77
3	Zone C	2638	2690	52	0.52	0.01	28	20	33	67

**Table 5:** Permeability and effective porosity of Sapphire wells (Sapphire-1-Sapphire-Da- Sapphire-Deep1 - Sapphire-DH).

Well name	$\:{\mathbf{k}}_{\varvec{mini}.}$	$\:{\mathbf{k}}_{\varvec{max}.}$	$\:{\mathbf{k}}_{\mathbf{avg}.}$	$\:\varvec{\Phi\:}$_mini_	$\:{\varvec{\Phi\:}}_{\varvec{max}.}$	$\:{\varvec{\Phi\:}}_{\mathbf{e}.\mathbf{avg}}$	Relationship	$\:{\mathbf{R}}^{2}$
Sapphire-1	0.01	117.78	15.09	0.03	0.33	0.18	$\:\text{k}=0.0874{\text{e}}^{25.055\varvec{\Phi\:}}$	0.9306
Sapphire-Da	51.47	525.35	227	0.06	0.27	0.15	$\:\text{k}=67.187{\text{e}}^{6.1716\varvec{\Phi\:}}$	0.4253
Sapphire-Deep1	1.18	950.30	136.34	0.1	0.35	0.28	$\:\text{k}=1.6279{\text{e}}^{12.804\varvec{\Phi\:}}$	0.936
Sapphire-Dh	1.08	141.73	16.88	0.1	0.34	0.18	$\:\text{k}=0.2075{\text{e}}^{21.296\varvec{\Phi\:}}$	0.9199

**Table 6:** Comparison of Reservoir properties between Sapphire Field and adjacent fields (Ha،py, Baltim, Abu Madi, and Sequoia).

Feature	Sapphire Field (current study)	Ha’py gas Field (Abdelbaset M. Abudeif et al., 2023)	Baltim gas Field^5^	Abu Madi Gas Field Al Khilala Field (Eysa, E. A. et al., 2015)	Sequoia Field (Hassan M. El Shayeb et al., 2020)
Porosity (Φ)	0.03 to 0.35 (Average 0.18)	0.28 to 0.36 (Average 0.18)	0.15 to 0.21 (Average unspecified)	0.1 to 0.25 (Average 0.18)	0.091 to 0.35 (Average 0.22)
Permeability (k)	0.01 to 117.78 mD. (Average 15.09)	0.01 to505.23 mD.	0.5 to 50 mD.	0.5 to 45 mD.	0.5 to 80 mD.
Gas Saturation ($\:{\mathbf{S}}_{\varvec{h}}$)	55–85%	50–75%	60–80%	80%	60–85.3%
Water Saturation ($\:{\mathbf{S}}_{\varvec{W}}$)	15–45%	10–60%	20–50%	10–40%	15–40%
Net Pay Thickness	0.46 m to 23.8 m	1 m to 20 m	6 m to 46 m	2 m to 24 m	10.06 m to 57.929 m
Lithology	Limestone, Sandstone, Shale	Sandstone, Shale	Sandstone, Shale	Sandstone, Shale, Small Limestone	Sandstone, Shale
Reservoir Quality	Good in some areas, but variability in water saturation and permeability	Moderate reservoir quality, higher water saturation	Good reservoir quality, higher water saturation in some areas	Excellent reservoir quality, lower water saturation	Good reservoir quality, higher gas saturation than Sapphire

#### Permeability-effective porosity relationship in the Sapphire-1 well

In the Sapphire-1 well, permeability spans from 0.01 to 117.78 millidarcies, with an average of 15.09 millidarcies. Effective porosity ranges from 0.03 to 0.33, with an average of 0.18 (Table 5). These parameters are key markers of reservoir quality, offering insights into fluid transmissibility and void spaces for hydrocarbon storage. The observed permeability variability highlights the heterogeneity of the reservoir rock, affecting fluid flow efficiency. Understanding these variations is essential for thorough reservoir characterization and effective management strategies for the Sapphire field (Fig. 27). These findings not only aid in academic understanding but also have practical implications for hydrocarbon recovery and field development planning. Permeability in the Sapphire-1 well ranges from 0.01 to 117.78 millidarcies, with an average of 15.09 millidarcies. Porosity ranges from 0.03 to 0.33, with a mean of 0.18. These values are crucial indicators of reservoir quality, showing fluid transmission capacity and void spaces for hydrocarbon storage. The permeability range indicates heterogeneity in reservoir rock, impacting fluid flow efficiency. Understanding these variations is crucial for effective reservoir management strategies in the Sapphire field. These insights not only contribute to geology but also optimize hydrocarbon recovery and development planning.

#### Permeability-effective porosity relationship in the Sapphire-Da well

In the Sapphire-Da well, permeability exhibits a range from 51.47 to 525.35 mD, with an average permeability value of 227 millidarcies. Concurrently, effective porosity demonstrates variability between 0.06 and 0.27, with an average effective porosity value of 0.15 (Table 5). These parameters stand as pivotal indicators of reservoir quality, providing nuanced insights into the rock’s fluid transmission capacity and the void spaces available for hydrocarbon storage. The discerned range in permeability values accentuates the inherent heterogeneity of the reservoir rock, influencing the efficiency of fluid flow within the geological formation. A comprehensive understanding of these variations is imperative for a thorough reservoir characterization, forming the foundational basis for effective reservoir management strategies in the Sapphire-Da (Fig. 28). These findings contribute not only to the academic understanding of geological principles but also bear practical implications for optimizing hydrocarbon recovery and guiding the strategic planning of field development.

#### Permeability-effective porosity relationship in the Sapphire-Deep1 well

In the Sapphire Deep-1 well, permeability ranges from 1.18 to 950.30 millidarcies, with an average of 136.34 millidarcies. Effective porosity varies from 0.1 to 0.35, with an average of 0.28 (Table 5). These parameters are key indicators for assessing reservoir quality, providing insights into fluid transmission capabilities and void spaces for hydrocarbon storage.The observed permeability variability highlights the heterogeneity of the reservoir rock, affecting fluid flow efficiency. Understanding these variations is essential for robust reservoir characterization and effective management strategies for Sapphire Deep-1 (Fig. 29). These findings enhance the academic understanding of geological principles and have practical implications for optimizing hydrocarbon recovery and field development planning.

#### Permeability-effective porosity relationship in the Sapphire-Dh well

In the Sapphire-Dh well, permeability ranges from 1.08 to 141.73 millidarcies, with an average permeability of 16.88 millidarcies. Simultaneously, effective porosity varies between 0.1 and 0.34, with an average effective porosity of 0.18 (Table 5). These parameters are critical indicators for assessing reservoir quality, providing detailed insights into the rock’s fluid transmission capabilities and the void spaces available for hydrocarbon storage. The observed variability in permeability underscores the inherent heterogeneity of the reservoir rock, influencing the efficiency of fluid flow within the geological formation. A comprehensive understanding of these variations is essential for robust reservoir characterization, forming the foundation for effective reservoir management strategies specific to the Sapphire-Dh (Fig. 30). These findings not only contribute to the academic understanding of geological principles but also have practical implications for optimizing hydrocarbon recovery and guiding strategic field development planning.

### Comparison between reservoir properties of Sapphire field and adjacent fields

Sapphire Field has promising reservoir characteristics, with a good balance of porosity and permeability, along with moderate to high gas saturation **(**Table 6**)**. However, the water saturation and permeability variability could affect recovery efficiency in certain zones. Ha’py Field shows slightly lower permeability and higher water saturation, which could present challenges for gas recovery. Its reservoir quality is generally moderate compared to Sapphire and the other fields. Baltim Field offers similar reservoir characteristics to Sapphire in terms of porosity and netpay thickness, but with higher water saturation in some zones, which may limit gas production efficiency. Abu Madi Field stands out with its lower water saturation, higher gas saturation, and good reservoir quality, which gives it a production advantage over Sapphire in terms of gas recovery. Sequoia Field has higher porosity and gas saturation than Sapphire, indicating that it may offer better hydrocarbon storage and production potential in certain zones, especially in terms of gas recovery. Impact on Sapphire: The adjacent fields— Ha’py, Baltim, Abu Madi, and Sequoia—provide valuable context for understanding the reservoir variability in the Sapphire field. Sapphire’s potential for hydrocarbon recovery can be enhanced by analyzing trends from these fields, particularly in terms of permeability, gas saturation, and water saturation, to develop effective reservoir management strategies. The field development planning for Sapphire can benefit from understanding how these fields optimize production efficiency and manage water saturation and gas recovery.

## Conclusions

- The Kafr El Sheikh Formation, identified within the study area, stands out as a highly promising reservoir rock, showcasing a nuanced range of petrophysical parameters.
- Effective porosity levels exhibit variability from 14 to 34%, while shale content presents a spectrum from 4 to 34%.
- Water saturation ranges from 15 to 45%, with hydrocarbon saturation demonstrating a noteworthy span between 55% and 85%.
- The net pay thickness extends from 0.46 m to 23.8 m.
- Lithological identification techniques confirm a composite composition of sandstone and shale within the formation.
- The developmental trajectory within the Sapphire Field underscores concentrated activity in the central and northwest-southwest regions, as indicated by discernible increases in effective porosity and hydrocarbon saturation, respectively.
- Capitalizing on the considerable hydrocarbon accumulation in the area, there exists substantial potential for further drilling of exploratory and development wells, offering opportunities to optimize productivity.
- The newly derived reservoir level contour maps serve as a foundational resource for constructing a comprehensive static and dynamic model for Sapphire Field, guiding strategic future well planning initiatives.

## Data Availability

The datasets used and/or analyzed during the current study available from the corresponding author on reasonable request.
